# Clinical course of pathologically confirmed corticobasal degeneration and corticobasal syndrome

**DOI:** 10.1093/braincomms/fcad296

**Published:** 2023-11-03

**Authors:** Ikuko Aiba, Yuichi Hayashi, Takayoshi Shimohata, Mari Yoshida, Yuko Saito, Koichi Wakabayashi, Takashi Komori, Masato Hasegawa, Takeshi Ikeuchi, Aya M Tokumaru, Keita Sakurai, Shigeo Murayama, Kazuko Hasegawa, Toshiki Uchihara, Yasuko Toyoshima, Yufuko Saito, Ichiro Yabe, Satoshi Tanikawa, Keizo Sugaya, Kentaro Hayashi, Terunori Sano, Masaki Takao, Motoko Sakai, Harutoshi Fujimura, Hiroshi Takigawa, Tadashi Adachi, Ritsuko Hanajima, Osamu Yokota, Tomoko Miki, Yasushi Iwasaki, Michio Kobayashi, Nobutaka Arai, Takuya Ohkubo, Takanori Yokota, Keiko Mori, Masumi Ito, Chiho Ishida, Masaharu Tanaka, Jiro Idezuka, Masato Kanazawa, Kenju Aoki, Masashi Aoki, Takafumi Hasegawa, Hirohisa Watanabe, Atsushi Hashizume, Hisayoshi Niwa, Keizo Yasui, Keita Ito, Yukihiko Washimi, Eiichiro Mukai, Akatsuki Kubota, Tatsushi Toda, Kenji Nakashima, J-VAC study group, Yuichi Hayashi, Takayoshi Shimohata, Mari Yoshida, Yuko Saito, Koichi Wakabayashi, Takashi Komori, Masato Hasegawa, Takeshi Ikeuchi, Aya M Tokumaru, Keita Sakurai, Shigeo Murayama, Kazuko Hasegawa, Toshiki Uchihara, Yasuko Toyoshima, Yufuko Saito, Ichiro Yabe, Satoshi Tanikawa, Keizo Sugaya, Kentaro Hayashi, Terunori Sano, Masaki Takao, Motoko Sakai, Harutoshi Fujimura, Hiroshi Takigawa, Tadashi Adachi, Ritsuko Hanajima, Osamu Yokota, Tomoko Miki, Yasushi Iwasaki, Michio Kobayashi, Nobutaka Arai, Takuya Ohkubo, Takanori Yokota, Keiko Mori, Masumi Ito, Chiho Ishida, Masaharu Tanaka, Jiro Idezuka, Masato Kanazawa, Kenju Aoki, Masashi Aoki, Takafumi Hasegawa, Hirohisa Watanabe, Atsushi Hashizume, Hisayoshi Niwa, Keizo Yasui, Keita Ito, Yukihiko Washimi, Eiichiro Mukai, Akatsuki Kubota, Tatsushi Toda, Kenji Nakashima, Shinya Tanaka, Kinya Ishikawa, Renpei Sengoku, Yasuhiro Sakashita, Tomoyasu Matsubara, Kimiko Inoue, Chiaki Mori, Tomoko Saito, Takahiko Tokuda, Hisanori Kowa, Seishi Terada, Hanae Nakashima-Yasuda, Yuko Kato-Motozaki, Kiyonobu Komai, Osamu Onodera, Akiyoshi Kakita, Hiroshi Shimizu, Mari Tada, Arifumi Matsumoto, Akio Kikuchi, Mutsufusa Watanabe, Masahisa Katsuno, Tosiaki Ieda, Meiko Hashimoto Maeda, Ikuko Aiba

**Affiliations:** Department of Neurology, NHO Higashinagoya National Hospital, Nagoya, Aichi 465-8620, Japan; Department of Neurology, Gifu University Graduate School of Medicine, Gifu 501-1194, Japan; Department of Neurology, Gifu University Graduate School of Medicine, Gifu 501-1194, Japan; Department of Neuropathology, Institute for Medical Science of Aging, Aichi Medical University, Nagakute, Aichi 480-1195, Japan; Department of Neuropathology (the Brain Bank for Aging Research), Tokyo Metropolitan Institute for Geriatrics and Gerontology, Itabashi, Tokyo 173-0015, Japan; Department of Pathology and Laboratory Medicine, National Center Hospital, National Center of Neurology and Psychiatry, Kodaira, Tokyo 187-8551, Japan; Department of Neuropathology, Hirosaki University Graduate School of Medicine, Hirosaki, Aomori 036-8562, Japan; Department of Laboratory Medicine and Pathology (Neuropathology), Tokyo Metropolitan Neurological Hospital, Fuchu, Tokyo 183-0042, Japan; Department of Brain & Neurosciences, Tokyo Metropolitan Institute of Medical Science, Setagaya, Tokyo 156-8506, Japan; Department of Molecular Genetics, Brain Research Institute, Niigata University, Chuo, Niigata 951-8585, Japan; Department of Diagnostic Radiology, Tokyo Metropolitan Institute for Geriatrics and Gerontology, Itabashi, Tokyo 173-0015, Japan; Department of Radiology, National Center for Geriatrics and Gerontology, Obu, Aichi 474-8511, Japan; Brain Bank for Neurodevelopmental, Neurological and Psychiatric Disorders, United Graduate School of Child Development, Osaka University, Suita, Osaka 565-0871, Japan; Department of Neurology and Neuropathology, Tokyo Metropolitan Institute for Geriatrics and Gerontology, Itabashi, Tokyo 173-0015, Japan; Department of Neurology, NHO Sagamihara National Hospital, Sagamihara, Kanagawa 252-0392, Japan; Neurology Clinic with Neuromorphomics Laboratory, Nitobe-Memorial Nakano General Hospital, Nakano, Tokyo 164-8607, Japan; Laboratory of Structural Neuropathology, Tokyo Metropolitan Institute of Medical Science, Setagaya, Tokyo 156-8506, Japan; Department of Neurology, Brain Disease Center Agano Hospital, Agano, Niigata 959-2221, Japan; Department of Pathology, Brain Research Institute, Niigata University, Chuo, Niigata 951-8585, Japan; Department of Neurology, NHO Higashinagoya National Hospital, Nagoya, Aichi 465-8620, Japan; Department of Neurology, Faculty of Medicine and Graduate School of Medicine, Hokkaido University, Sapporo, Hokkaido 060-8638, Japan; Institute for Chemical Reaction Design and Discovery (WPI-ICReDD), Hokkaido University, Sapporo, Hokkaido 001-0021, Japan; Department of Neurology, Tokyo Metropolitan Neurological Hospital, Fuchu, Tokyo 183-0042, Japan; Department of Neurology, Tokyo Metropolitan Neurological Hospital, Fuchu, Tokyo 183-0042, Japan; Department of Laboratory Medicine, National Center Hospital, National Center of Neurology and Psychiatry, Kodaira, Tokyo 187-8551, Japan; Department of Laboratory Medicine, National Center Hospital, National Center of Neurology and Psychiatry, Kodaira, Tokyo 187-8551, Japan; Department of Neurology, NHO Suzuka National Hospital, Suzuka, Mie 513-8501, Japan; Department of Neurology, NHO Osaka Toneyama Medical Center, Toyonaka, Osaka 560-8552, Japan; Division of Neurology, Department of Brain and Neurosciences, Faculty of Medicine, Tottori University, Yonago, Tottori 683-8503, Japan; Division of Neuropathology, Department of Brain and Neurosciences, Faculty of Medicine, Tottori University, Yonago, Tottori 683-8503, Japan; Division of Neurology, Department of Brain and Neurosciences, Faculty of Medicine, Tottori University, Yonago, Tottori 683-8503, Japan; Department of Psychiatry, Kinoko Espoir Hospital, Kasaoka, Okayama 714-0071, Japan; Department of Neuropsychiatry, Okayama University Graduate School of Medicine, Dentistry and Pharmaceutical Sciences, Kita, Okayama 700-8558, Japan; Department of Psychiatry, Kinoko Espoir Hospital, Kasaoka, Okayama 714-0071, Japan; Department of Neuropsychiatry, Okayama University Graduate School of Medicine, Dentistry and Pharmaceutical Sciences, Kita, Okayama 700-8558, Japan; Department of Neuropathology, Institute for Medical Science of Aging, Aichi Medical University, Nagakute, Aichi 480-1195, Japan; Department of Neurology, NHO Akita National Hospital, Yurihonjo, Akita 018-1393, Japan; Laboratory of Neuropathology, Tokyo Metropolitan Institute of Medical Science, Setagaya, Tokyo 156-8506, Japan; Department of Neurology and Neurological Sciences, Tokyo Medical and Dental University, Bunkyo, Tokyo 113-8519, Japan; Department of Neurology and Neurological Sciences, Tokyo Medical and Dental University, Bunkyo, Tokyo 113-8519, Japan; Department of Neurology, Oyamada Memorial Spa Hospital, Yokkaichi, Mie 512-1111, Japan; Department of Neurology, Oyamada Memorial Spa Hospital, Yokkaichi, Mie 512-1111, Japan; Department of Neurology, NHO Iou National Hospital, Kanazawa, Ishikawa 920-0192, Japan; Department of Psychiatry, Mishima Hospital, Nagaoka, Niigata 940-2302, Japan; Department of Neurology, Ojiya Sakura Hospital, Ojiya, Niigata 947-0041, Japan; Department of Neurology, Clinical Neuroscience Branch, Brain Research Institute, Niigata University, Chuo, Niigata 951-8585, Japan; Department of Neurology, Brain Disease Center Agano Hospital, Agano, Niigata 959-2221, Japan; Department of Neurology, Tohoku University Graduate School of Medicine, Sendai, Miyagi 980-8574, Japan; Department of Neurology, Tohoku University Graduate School of Medicine, Sendai, Miyagi 980-8574, Japan; Department of Neurology, Fujita Health University School of Medicine, Toyoake, Aichi 470-1192, Japan; Department of Clinical Research Education, Nagoya University Graduate School of Medicine, Nagoya, Aichi 466-8550, Japan; Department of Neurology, Kariya Toyota General Hospital, Kariya, Aichi 448-8505, Japan; Department of Neurology, Japanese Red Cross Aichi Medical Center Nagoya Daini Hospital, Nagoya, Aichi 466-8650, Japan; Department of Neurology, Hekinan Municipal Hospital, Hekinan, Aichi 447-8502, Japan; Department of Geriatrics and Gerontology, National Center for Geriatrics and Gerontology, Obu, Aichi 474-8511, Japan; Department of Neurology, Aichi-pref Saiseikai Rehabilitation Hospital, Nagoya, Aichi 451-0052, Japan; Department of Neurology, Graduate School of Medicine, The University of Tokyo, Bunkyo, Tokyo 113-8655, Japan; Department of Neurology, Graduate School of Medicine, The University of Tokyo, Bunkyo, Tokyo 113-8655, Japan; Department of Neurology, NHO Matsue Medical Center, Matsue, Shimane 690-8556, Japan

**Keywords:** corticobasal degeneration, corticobasal syndrome, clinical course, pathology, diagnosis

## Abstract

The clinical presentation of corticobasal degeneration is diverse, while the background pathology of corticobasal syndrome is also heterogeneous. Therefore, predicting the pathological background of corticobasal syndrome is extremely difficult. Herein, we investigated the clinical findings and course in patients with pathologically, genetically and biochemically verified corticobasal degeneration and corticobasal syndrome with background pathology to determine findings suggestive of background disorder. Thirty-two patients were identified as having corticobasal degeneration. The median intervals from the initial symptoms to the onset of key milestones were as follows: gait disturbance, 0.0 year; behavioural changes, 1.0 year; falls, 2.0 years; cognitive impairment, 2.0 years; speech impairment, 2.5 years; supranuclear gaze palsy, 3.0 years; urinary incontinence, 3.0 years; and dysphagia, 5.0 years. The median survival time was 7.0 years; 50% of corticobasal degeneration was diagnosed as corticobasal degeneration/corticobasal syndrome at the final presentation. Background pathologies of corticobasal syndrome (*n* = 48) included corticobasal degeneration (33.3%), progressive supranuclear palsy (29.2%) and Alzheimer’s disease (12.5%). The common course of corticobasal syndrome was initial gait disturbance and early fall. In addition, corticobasal degeneration–corticobasal syndrome manifested behavioural change (2.5 years) and cognitive impairment (3.0 years), as the patient with progressive supranuclear palsy–corticobasal syndrome developed speech impairment (1.0 years) and supranuclear gaze palsy (6.0 years). The Alzheimer’s disease–corticobasal syndrome patients showed cognitive impairment (1.0 years). The frequency of frozen gait at onset was higher in the corticobasal degeneration–corticobasal syndrome group than in the progressive supranuclear palsy–corticobasal syndrome group [*P* = 0.005, odds ratio (95% confidence interval): 31.67 (1.46–685.34)]. Dysarthria at presentation was higher in progressive supranuclear palsy–corticobasal syndrome than in corticobasal degeneration–corticobasal syndrome [*P* = 0.047, 6.75 (1.16–39.20)]. Pyramidal sign at presentation and personality change during the entire course were higher in Alzheimer’s disease–corticobasal syndrome than in progressive supranuclear palsy–corticobasal syndrome [*P* = 0.011, 27.44 (1.25–601.61), and *P* = 0.013, 40.00 (1.98–807.14), respectively]. In corticobasal syndrome, decision tree analysis revealed that ‘freezing at onset’ or ‘no dysarthria at presentation and age at onset under 66 years in the case without freezing at onset’ predicted corticobasal degeneration pathology with a sensitivity of 81.3% and specificity of 84.4%. ‘Dysarthria at presentation and age at onset over 61 years’ suggested progressive supranuclear palsy pathology, and ‘pyramidal sign at presentation and personality change during the entire course’ implied Alzheimer’s disease pathology. In conclusion, frozen gait at onset, dysarthria, personality change and pyramidal signs may be useful clinical signs for predicting background pathologies in corticobasal syndrome.

## Teaser

Aiba *et al*. elucidated that detailed clinical findings and course of 32 patients with corticobasal degeneration and 48 patients with corticobasal syndrome with background pathology. They uncovered that frozen gait at onset, dysarthria, personality changes and pyramidal signs may be useful clinical signs for predicting background pathologies in corticobasal syndrome.

## Graphical Abstract

**Graphical Abstract:**
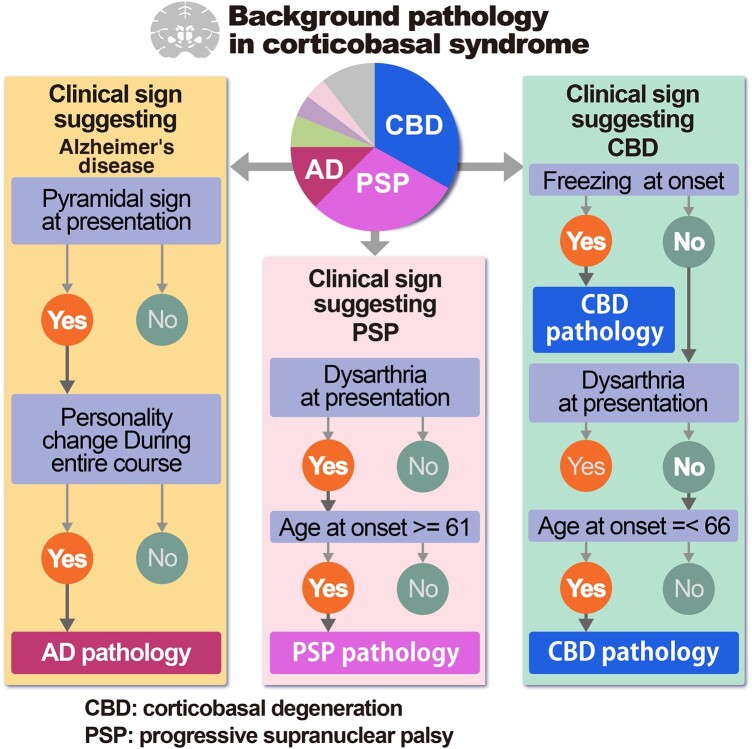

See I. McGeachan and King (https://doi.org/10.1093/braincomms/fcad321) for a scientific commentary on this article.

## Introduction

Corticobasal degeneration (CBD) is a rare neurodegenerative disorder characterized by neuronal loss and the predominance of hyperphosphorylated 4-repeat (4R) tau deposition in various brain regions.^1–4^ Recently, the 3D structure of tau filaments of CBD was identified using cryo-electron microscopic analysis.^5^ The protofilament structure in CBD is distinct from other 4R tauopathies, such as progressive supranuclear palsy (PSP). Corticobasal syndrome (CBS) is the classic phenotype of CBD, presenting with asymmetric apraxia, rigidity, dystonia, myoclonus, cortical sensory loss and alien limb.^1,6,7^ However, CBD can manifest in several clinical syndromes, including PSP syndrome (PSPS),^8–12^ frontal behavioural–spatial syndrome (FBS),^8–13^ non-fluent/agrammatic variant of primary progressive aphasia (naPPA)^8–10^ and Alzheimer’s-like dementia.^8,9,12,13^ Conversely, pathological backgrounds of CBS are broad.^9,10,14–23^ The most frequent cause of CBS is CBD,^7,10,15,18–22,24,25^ followed by Alzheimer’s disease (AD),^7,11,14,15,18–20,26–29^ PSP^7,10,11,15,18–20,23^ and many other diseases. CBD and PSP are both 4R tauopathies and have substantial overlap that will likely preclude differentiating of the two, clinically, in some cases. Therefore, the rate of correct diagnosis of CBD in daily life is extremely low.^8,9,13,19^

Several diagnostic criteria have been proposed.^7,30–35^ In 2013, Armstrong *et al*. proposed new clinical diagnostic criteria for CBD (Armstrong’s criteria),^9^ which according to a subsequent validation study did not have very high sensitivity and had low specificity.^14,17^ However, these articles^14,17^ failed to consider the exclusion criteria (e.g. AD biomarkers) when assessing the CBD criteria.^36^ Previous studies have revealed some clinical findings suggestive of non-CBD pathology in CBS.^27,37,38^ On the other hand, no clinical signs suggesting CBD pathology in CBS have been reported.^14,17^ It is unknown whether the course of CBS differs according to the background pathology. Hence, detecting CBD pathology in CBS is extremely challenging.

Therefore, we analysed the clinical findings and course of pathologically confirmed CBD and CBS to determine whether the clinical signs and course suggestive of CBD pathology can be detected in CBS.

## Materials and methods

### Identification of patients

We conducted a Japanese validation study of the consensus criteria for CBD diagnosis (J-VAC study) within the framework of the Research Committee of CNS Degenerative Diseases, Research on Policy Planning and Evaluation for Rare and Intractable Diseases, Health, Labour, and Welfare Sciences Research Grants, the Ministry of Health, Labour and Welfare, Japan. The J-VAC study is a retrospective study of a pathology cohort. Forty-eight centres were involved in this study including 15 pathological centres and 32 clinical facilities. Most of the centres in the J-VAC study were facilities with a background in movement disorders, wherein neurology specialists made the clinical diagnosis. The pathological diagnosis data by neuropathologists at each institution between 1996 and 2018 were retrieved. The neuropathologists at each institution pathologically diagnosed patients with CBD using frozen tissues, and those with a clinical diagnosis of CBD or CBS, but without CBD pathology, as per Alexander *et al.*’s^14^ validation study of Armstrong’s criteria, termed ‘CBD mimics’. The pathological centres requested the clinical facility to fill out a clinical information chart for the pathologically diagnosed patients. Patients with insufficient clinical data were excluded from this study. Informed consent was obtained from the patients as an opt-out on the website. The study was approved by the Ethics Committee of the National Hospital Organization Higashinagoya National Hospital (#27-8), and each institute was in accordance with the Declaration of Helsinki.

### Evaluation of patients with pathological diagnosis of CBD

#### Pathological analysis

To standardize the pathological diagnosis and elucidate the pathological features of Japanese patients with CBD, the pathological findings were retrospectively reviewed by an independent group of neuropathologists (T.K., Y.S., K.W. and M.Y.) supported by the Brain Bank Committee of the Japanese Society of Neuropathology.

Formalin-fixed, paraffin-embedded glass slide specimens were collected using haematoxylin–eosin, Klüver–Barrera, Gallyas–Braak (G-B) silver methods, phosphorylated tau (AT8) and amyloid β protein immunohistochemistry.

These specimens were sent to neuropathologists and reviewed independently, blinded to clinical information, while filling out a pathological diagnostic datasheet (Supplementary Table 1). The pathologic diagnostic datasheet was based on the neuropathologic criteria for CBD, as proposed by Dickson *et al*.^2^ Finally, we discussed whether the neuropathological diagnosis is relevant to CBD along with other additional pathological aspects in all cases.

#### Genetic analysis

Genomic DNA was extracted from frozen brain tissues using a standard procedure. Mutational analysis was performed by sequencing both strands of all polymerase chain reaction-amplified coding exons and flanking the intronic sequences of *MAPT*, as previously reported.^39^

#### Biochemical analysis

Biochemical analysis of the tau from either the frontal or temporal lobe cortex accumulated in the brains of patients was conducted by investigating the banding pattern of C-terminal fragments of tau on the immunoblot with anti-tau antibodies, as previously reported.^40^ Sarkosyl insoluble tau was prepared from ∼0.5 g frozen tissues essentially, as has been previously described.^41,42^

### Clinical data collection and analysis of patients with CBD pathology

Clinical evaluations were performed on patients with pathologically, genetically and biochemically verified CBD during the central review, and each patient’s clinical information was retrospectively analysed. The evaluation items included initial signs and symptoms, major CBD signs and symptoms (at the time of examination and during the entire clinical course) based on the definitions of Armstrong’s criteria^9^ and some relevant diagnostic criteria.^30,43,44^ When data were abstracted, items were considered as present or absent only if described.

We analysed the intervals between the initial symptoms and key clinical milestones. Clinical features observed in more than half of the patients were selected as key milestones. We also examined whether the course differed according to the clinical type.

### Evaluation of patients with clinical diagnosis of CBD or CBS without CBD pathology (CBD mimics)

#### Clinical data collection and analysis

In patients with CBD mimics, clinical evaluation was further performed using the same items as those with pathologically verified CBD. Moreover, these patients had met the Mayo Clinic^7^ or the Cambridge^35^ diagnostic criteria for CBS.

#### Comparison analysis between CBD and CBD mimics

We compared the frequency of clinical symptoms and signs of CBD with those of CBD mimics to identify the former at three clinical points in their clinical course: at onset, at presentation or during the entire course. In addition, we compared the interval from the initial symptoms to the key milestones between CBD and CBD mimics.

### Evaluation of patients with CBS

#### Background pathology of CBS

We defined CBS as patients whose final clinical diagnosis was CBD or CBS. We analysed the background pathology of patients with CBS, including CBD–CBS (patients with CBD pathology with a clinical diagnosis of CBS or CBD) and CBD mimics and their clinical information.

#### Comparison analysis among CBD–CBS, PSP–CBS and AD–CBS

We compared the frequency of symptoms and signs of CBD–CBS with those of PSP–CBS or AD–CBS (AD pathology with a clinical diagnosis of CBS) at onset, presentation or during the entire course. Moreover, we compared the interval from the initial symptoms to the key milestones of CBD–CBS with those of PSP–CBS or AD–CBS.

A decision tree analysis was performed using the classification and regression tree method with CBD, PSP and AD as the dependent variables and sex, age at onset and significant findings as the independent variables; cross-validation was further performed.

### Statistical analysis

Statistical analysis was performed using SPSS software version 22 (IBM, Inc., Armonk, NY, USA). Data were assessed using Fisher’s exact test and odds ratio [OR; 95% confidence interval (95% CI)] to compare the frequency of symptoms and signs. Survival was calculated using Kaplan–Meier analysis, and the log-rank test was used for comparisons. *P* < 0.05 was considered statistically significant.

## Results

### Patients with pathological diagnosis of CBD

#### Confirmation of CBD

**Figure 1:**
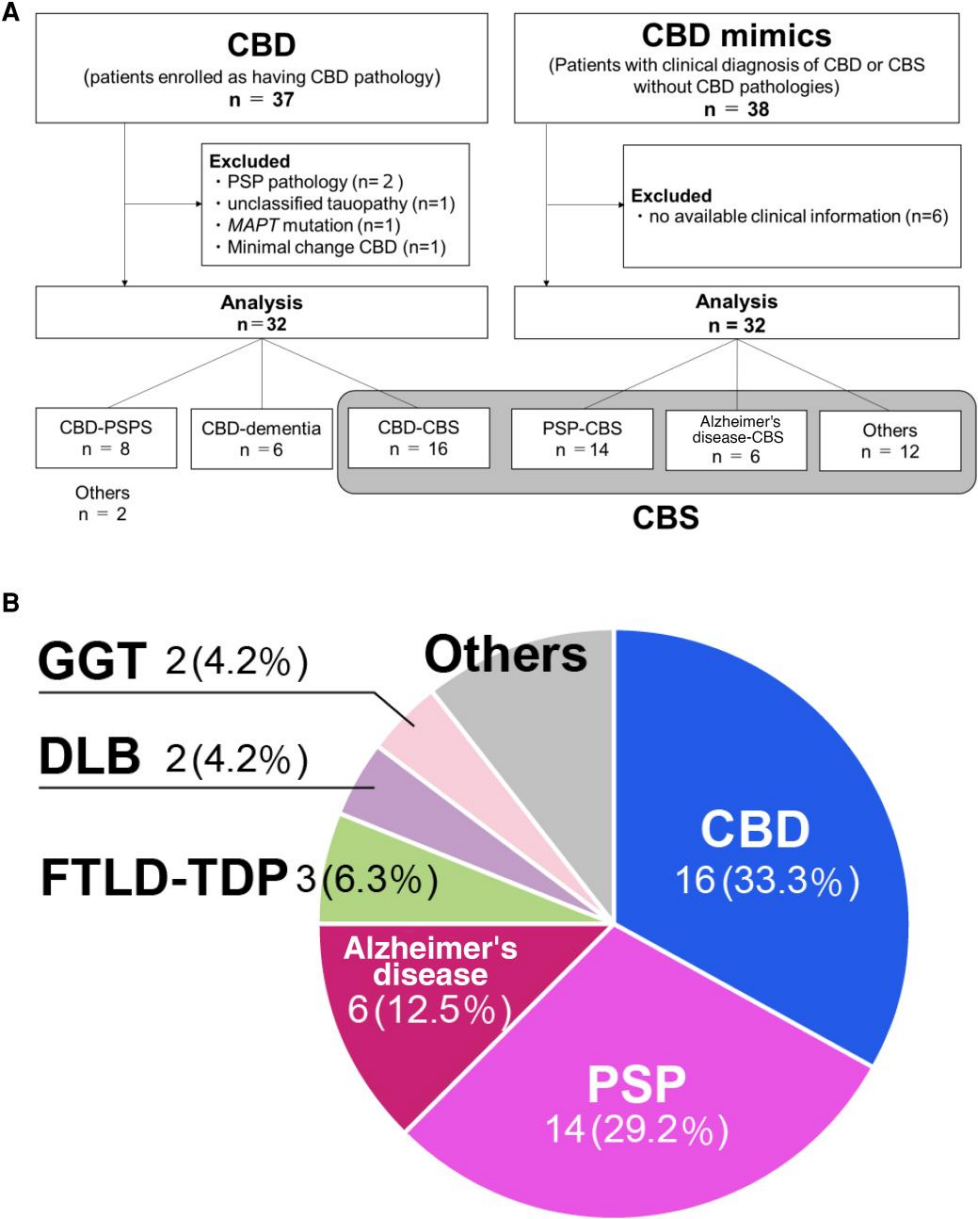
**Analytic flow and background pathology in CBS.** (**A**) Analytic flow for CBD and CBD mimics. CBD–PSPS, corticobasal degeneration (CBD) presented progressive supranuclear palsy (PSP) syndrome; CBD–CBS, CBD presented corticobasal syndrome (CBS); PSP–CBS, PSP presented CBS; AD–CBS, Alzheimer’s disease (AD) presented CBS. (**B**) The background pathology of CBS. CBS, corticobasal syndrome; CBD, corticobasal degeneration; PSP, progressive supranuclear palsy; FTLD-TDP, frontotemporal dementia (FTLD) with TDP-43 pathology; DLB, dementia with Lewy bodies; GGT, globular glial tauopathy. The background pathology of CBS includes various tauopathies. CBD was most common, followed by PSP, AD FTLD-TDP, DLB, and GGT. Others included FTLD fused in sarcoma (FUS) (*n* = 1), glioblastoma (*n* = 1), Pick’s disease (*n* = 1), prion disease (*n* = 1) and non-specific pathological changes (*n* = 1).

Thirty-seven patients with CBD pathology diagnosed by a neuropathologist at each institution were enrolled in our study (Fig. 1A). After central pathological, genetic and biochemical verification of the 37 patients, a consensus meeting for the J-VAC study was held online in September 2020. Finally, we identified genetic, biochemical and pathologically diagnosed CBD.

The results of pathological, genetic and biochemical analyses are shown in Supplementary Table 2. The essential pathological changes in the CBD were cortical and subcortical tau pathologies. The pathological hallmarks were numerous with the widespread distribution of threads and presence of astrocytic plaques, which were both positive for G–B silver staining and phosphorylated tau immunohistochemistry. Astrocytic plaques are important disease-specific structures, and we confirmed typical and sufficient astrocytic plaques in all cases. The density of astrocytic plaques was relatively decreased in severe degenerative cortical areas with long disease duration. Ballooned neurons (BN) are essential to CBD but not specific to CBD. The frequency of BN was variable (Supplementary Fig. 1).

Five patients were excluded: two with PSP pathological changes (Patient nos. 1 and 25), one with atypical 4R tau pathology and Lewy body pathology (Patient no. 30), one with extremely minimal pathological changes such as CBD (Patient no. 5) and one with *MAPT* mutation (Patient no. 27; Supplementary Table 2). Finally, we agree that the pathological diagnosis of CBD was appropriate for the present study in 32 patients.

### Clinical features of patients with CBD pathology (CBD)

#### Demographic data

The CBD patients were 16 men and 16 women. The mean age at onset was 65.4 years; mean duration from symptom onset to presentation was 3.4 years; and mean age at death was 73.0 years (Table 1). None of the patients had any family history.

**Table 1:** Demographic data in patients with confirmed CBD pathology (CBD) and clinical diagnosis of CBS or CBD with non-CBD pathology (CBD mimics)

Feature	CBD	CBD mimics	*P*-value (95% CI)
	*n* = 32	*n* = 32	
Age at onset, years	65.4 ± 8.1 (45–83)	66.0 ± 10.4 (33–86)	0.820 (−5.17 to 4.13)
M:F ratio	16:16	22:10	0.203, OR: 0.45 (0.45–1.26)
Time at presentation since onset of symptoms, years	3.4 ± 2.4 (0–9)	3.7 ± 3.1 (0–12)	0.667 (−1.69 to 1.09)
Age at death, years	73.0 ± 8.2 (50–87)	74.3 ± 10.0 (48–93)	0.578 (−5.84 to 3.30)
Duration of disease, years	7.8 ± 3.1 (3–17)	9.6 ± 5.5 (3–31)	0.108 (−4.02–0.41)

Data are presented as mean ± SD (range). M, male; F, female; OR, odds ratio; 95% CI, 95% confidence interval.

#### Initial symptoms and signs in CBD

The most frequent initial sign of CBD was gait disturbance (74%), followed by bradykinesia (64%). Clumsy limbs, falls and amnesia were the next most frequent symptoms, but these were less than half. In terms of the type of gait disturbance, slow gait was the most common (57%), followed by unstable gait (48%), frozen gait (39%) and short-step gait (36%; Supplementary Table 3).

### Frequency of clinical signs and symptoms in CBD

#### Motor features

Supplementary Table 4 shows the frequency of the clinical features in patients with CBD. The most common motor features were limb rigidity or bradykinesia (87%), followed by gait disturbance (80%) and postural instability or falls (65%). These findings had increased by the death of >90% of patients with CBD. Regarding the gait disorders, slow and unstable gait was observed around in 60% at presentation and >90% during the entire course. A frozen gait was observed in more than half of their lives. However, only 42% had dystonia and 25% had myoclonus during the entire course, in terms of asymmetric presentation, 65% of CBD had asymmetric limb rigidity or bradykinesia, 31% had asymmetric dystonia and 14% had asymmetric myoclonus (Supplementary Table 4).

#### Higher cortical features

The most common higher cortical feature was cognitive impairment (90%), followed by executive dysfunction (84%). Behavioural changes were also observed in more than half of the entire course. Limb apraxia was only observed in 29% of CBD at presentation and in less than half (48%) of their lives. Cortical sensory loss (21%) and alien limb signs (7%) were uncommonly observed. Asymmetric presentations were infrequent (Supplementary Table 4).

#### Other features

Among other features, urinary incontinence was observed in over 80% and supranuclear gaze palsy or decreased velocity of vertical saccades in over 60% of patients with CBD during the entire course. Speech and language impairments were also observed in 75%; dysarthria was the most frequent feature (Supplementary Table 4).

### Clinical diagnosis in CBD

At the initial presentation, 22% of CBD were diagnosed as CBD or CBS and 50% at the final presentation. The second most common clinical diagnosis was PSP (19% at initial presentation and 25% at the final presentation, respectively); 13% of CBD were diagnosed with Alzheimer’s disease at the initial presentation and 9% at the final presentation. Patients who were given a diagnosis of dementia, including AD, frontotemporal dementia (FTD), dementia with Lewy bodies (DLB) and Pick’s disease were 31% at initial diagnosis and 22% at the final diagnosis (Supplementary Table 5).

The most common clinical department for treatment was neurology at the initial stage (78%) and at the end stage (91%).

### Interval from the initial symptoms to a key milestone in CBD

**Figure 2:**
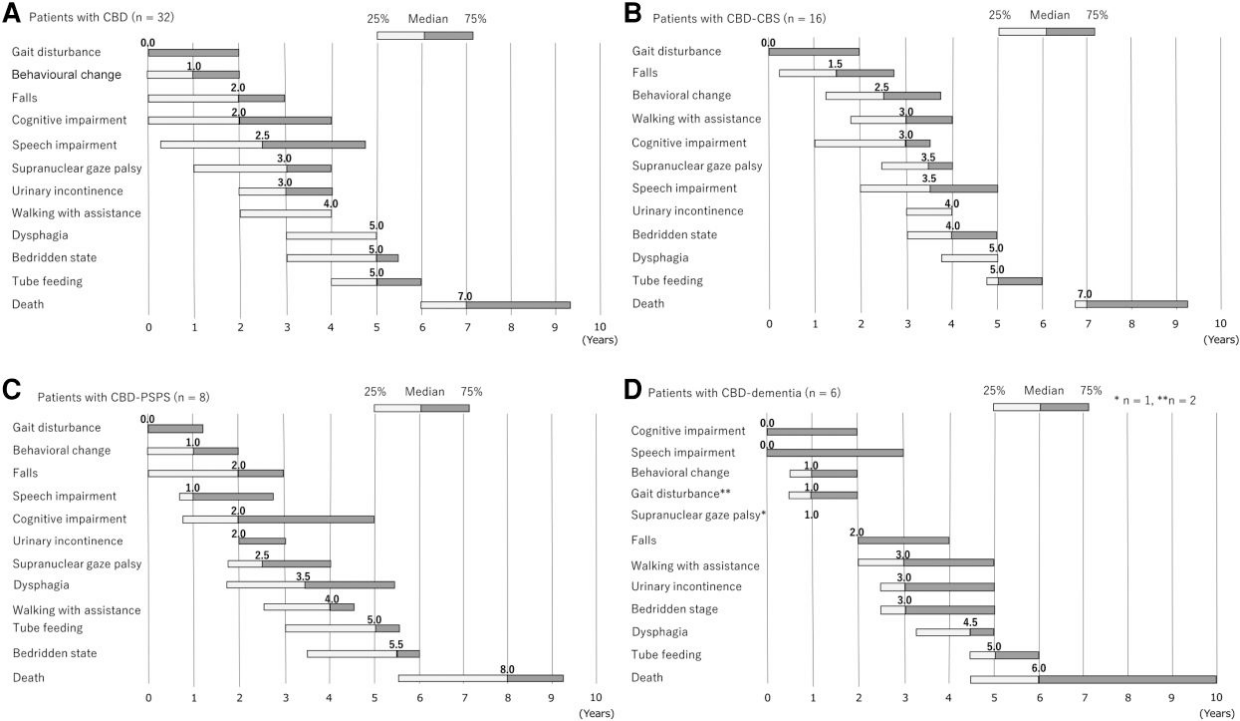
Interval from the initial symptom to key milestones in CBD. (**A**) Patients with CBD. (**B**) Patients with CBD–CBS. (**C**) Patients with CBD–PSPS. (**D**) Patients with CBD–dementia. CBD, corticobasal degeneration; CBS, corticobasal syndrome; PSPS, progressive supranuclear palsy syndrome.

The median intervals from the initial symptoms to the onset of key milestones were as follows: gait disturbance, 0.0 years; behavioural changes, 1.0 years; falls, 2.0 years; cognitive impairment, 2.0 years; speech impairment, 2.5 years; supranuclear gaze palsy, 3.0 years; urinary incontinence, 3.0 years; walking with assistance, 4.0 years; dysphagia, 5.0 years; and a bedridden state, 5.0 years (Fig. 2A).

We divided the patients into three subclinical types depending on the final clinical diagnosis (CBD–CBS: the final clinical diagnosis was CBS or CBD; CBD–PSPS: the diagnosis was PSP; and CBD–dementia: the diagnosis was AD or FTD, respectively). Behavioural changes, speech impairment, urinary incontinence and dysphagia tended to appear earlier in patients with CBD–PSPS than in those with CBS. In CBD–dementia, cognitive and speech impairments appeared earlier than in CBS and PSPS (Fig. 2B–D).

### Survival and cause of death in CBD

The Kaplan–Meier survival curve for CBD is shown in Supplementary Fig. 2A. The median survival time was 7.0 years. Age at onset, any symptoms at onset and clinical phenotypes were not related to the survival time. In women, the survival time was significantly longer than in men (7.0 years in men and 9.0 years in women, *P* = 0.046; Supplementary Fig. 2B). The most common cause of death was pneumonia (66%).

### Clinical phenotype of Armstrong’s criteria in CBD

**Figure 3:**
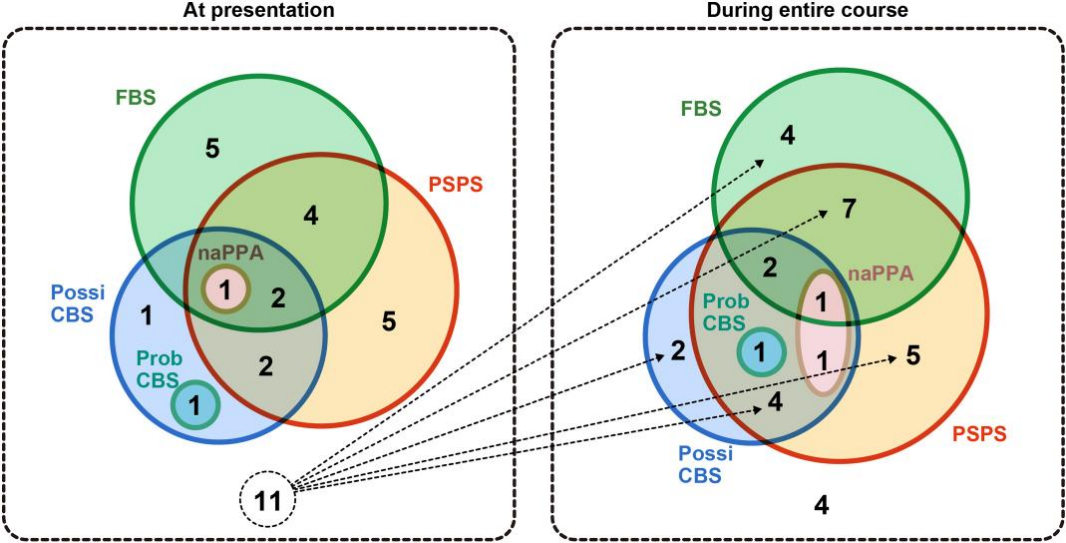
Combination of clinical phenotype in CBD. Each number is a number of patients who met the item of Armstrong’s criteria. The most common clinical type was PSPS (48 or 84% at the presentation or during the entire course, respectively), followed by FBS (48 or 64%) and possible CBS (32 or 46%) using Armstrong’s criteria. Many patients with CBD met the multiple clinical phenotypes of the Armstrong criteria. Conversely, 11 patients at presentation and four during the course did not meet any clinical criterion. One patient data during the entire course were unavailable. CBD, corticobasal degeneration; PSPS, progressive supranuclear palsy syndrome; FBS, frontal behavioural–spatial syndrome; naPPA, non-fluent/agrammatic variant of primary progressive aphasia; Possi CBS, possible corticobasal syndrome; Prob CBS, probable corticobasal syndrome.

The clinical types are shown in Table 2, and their combinations are shown in Fig. 3. At presentation, only 11 of 32 individuals with CBD had completed assessments for all the symptoms and signs in the Armstrong’s criteria, and nine further patients had completed assessments during the entire course of the study owing to the retrospective nature, as described above. Although the present study is retrospective and limited in scope as described above, the most common clinical type was PSPS (48 or 84%, at presentation or during the entire course, respectively), followed by FBS (48 or 64%) and possible CBS (32 or 46%) using Armstrong’s criteria.^9^ Only one patient exhibited probable CBS (4%), both at presentation and during the entire course; patients who met the criteria for naPPA were 4 or 10%. Eleven patients at presentation and four during the entire course exhibited no clinical type (Fig. 3).

**Table 2:** Frequency of clinical type of Armstrong’s criteria in patients with CBD

	CBD (*n* = 32)	
Clinical type	At presentation	During the entire course
Probable CBS	1/28 (4)	1/25 (4)
Possible CBS	7/22 (32)	11/23 (46)
FBS	12/25 (48)	14/22 (64)
naPPA	1/27 (4)	2/21 (10)
Progressive supranuclear palsy syndrome	13/27 (48)	21/25 (84)

Data are presented as *n* (%). CBD, corticobasal degeneration; CBS, corticobasal syndrome; FBS, frontal behavioural–spatial syndrome; naPPA, non-fluent/agrammatic variant of primary progressive aphasia.

### Patients with clinical diagnosis of CBD or CBS without CBD pathologies (CBD mimics)

#### Background of pathology

Thirty-eight patients with CBD mimics were enrolled; however, six patients were excluded because of no available clinical information (Fig. 1A). Supplementary Table 6 shows that patients with CBD mimics involved PSP (*n* = 14), including a patient with PSP with glioblastoma, AD (*n* = 6),^28,29^ frontotemporal lobar degeneration with TDP-43 pathology (*n* = 3), globular glial tauopathy (*n* = 2), DLB (*n* = 2)^45^ and others.^46,47^

### Clinical evaluation

#### Initial symptoms and signs in CBD mimics

In patients with CBD mimics, the most frequent initial sign was gait disturbance (69%), followed by clumsy limbs (63%), speech disturbances (55%) and bradykinesia (54%). In terms of the type of gait disturbance, unstable gait was the most common (46%). However, a frozen gait was not observed as an initial symptom (Supplementary Table 3).

### Frequency of clinical signs and symptoms in CBD mimics

#### Motor features

During the entire course, limb rigidity or bradykinesia, (100%) gait disturbance (97%) and postural instability or falls (89%) were frequently observed in patients with CBD mimics. Conversely, 58% of the patients had dystonia and 37% had myoclonus (Supplementary Table 4).

At presentation, 84% of them had limb rigidity or bradykinesia, and 77% presented gait disturbance. In terms of asymmetric presentation, 75% of CBD mimics had asymmetric limb rigidity or bradykinesia, 39% had asymmetric dystonia and 13% had asymmetric myoclonus (Supplementary Table 4).

#### Higher cortical features

For higher cortical features, both general cognitive impairment and frontal executive dysfunction were observed in more than 95% of patients, followed by limb apraxia (73%) during the entire course. However, 42% of CBD mimics presented behavioural changes, 39% had cortical sensory loss and 36% presented personality change during the entire course. An asymmetric alien limb sign was not observed (Supplementary Table 4).

#### Other features

In other features, urinary incontinence along with speech and language impairment was over 80%, supranuclear gaze palsy or decreased velocity of vertical saccades, and dysarthria was observed in over 75%, and the pyramidal sign was in 60% of those patients during the entire course (Supplementary Table 4).

### Interval from the initial symptoms to a key milestone in CBD mimics

**Figure 4:**
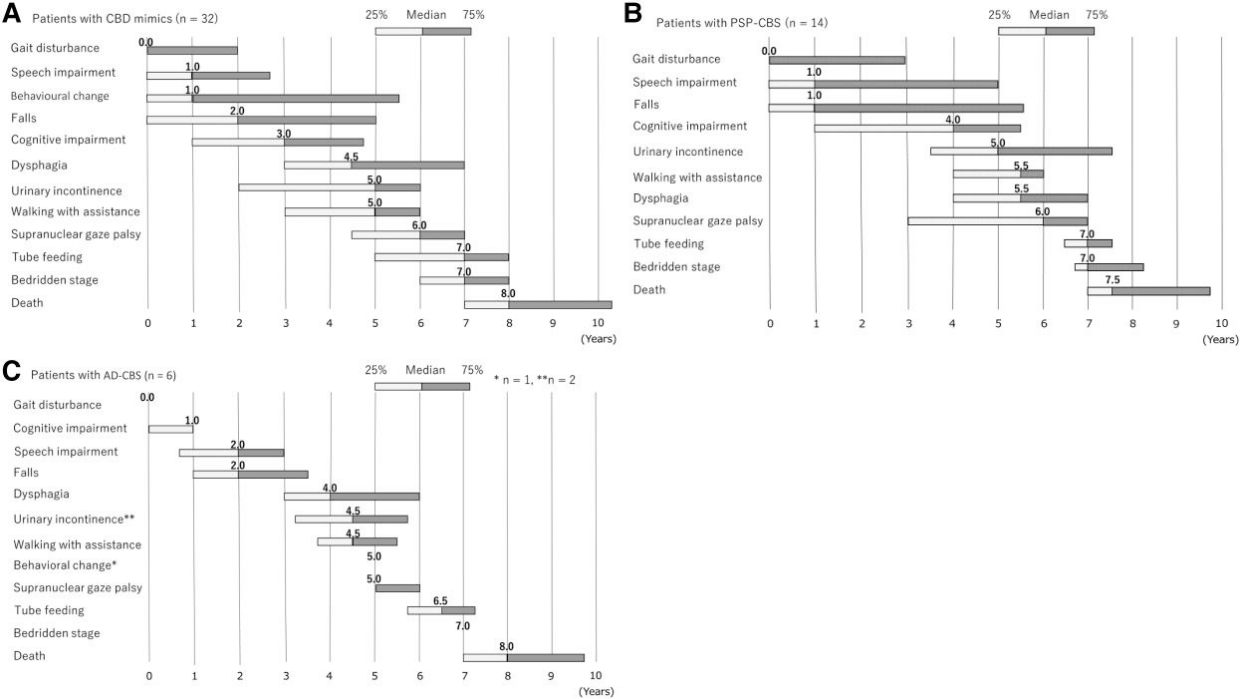
Interval from the initial symptom to key milestones in CBD mimics. (**A**) Patients with CBD mimics. (**B**) Patients with PSP–CBS. (**C**) Patients with AD–CBS. CBD, corticobasal degeneration; CBS, corticobasal syndrome; PSP, progressive supranuclear palsy.

The median intervals from the initial symptoms to the onset of key clinical milestones in CBD mimics were as follows: gait disturbance, 0.0 years; speech impairment, 1.0 years; behavioural changes, 1.0 years; falls, 2.0 years; cognitive impairment, 3.0 years; dysphagia, 4.5 years; urinary incontinence, 5.0 years; walking with assistance, 5.0 years; supranuclear gaze palsy, 6.0 years; and a bedridden state, 7.0 years (Fig. 4A).

### Comparison analysis between CBD and CBD mimics

#### Clinical symptoms and signs

We compared the initial symptoms of CBD with those of CBD mimics to identify the former. The frequencies of frozen gait [*P* = 0.002, OR (95% CI): 29.48 (1.59–546.35)] and tremor [*P* = 0.014, OR (95% CI): 5.70 (1.40–23.30)] were higher in the CBD group (Supplementary Table 3).

The frequencies of limb apraxia [*P* = 0.031, OR (95% CI): 3.64 (1.18–11.18)], dysarthria [*P* = 0.030, OR (95% CI): 3.82 (1.25–11.68)] and pyramidal signs [*P* = 0.034, OR (95% CI): 3.50 (1.17–10.54)] were higher in CBD mimics at presentation. The frequency of asymmetric limb rigidity or bradykinesia was higher in the CBD mimics [*P* = 0.031, OR (95% CI): 5.13 (1.27–20.81)]; however, the frequency of tremors was higher in the CBD during the entire course [*P* = 0.042, OR (95% CI): 3.67 (1.07–12.55); Supplementary Table 4].

#### Interval from the initial symptoms to a key milestone

Speech impairment tended to appear earlier in patients with CBD mimics than in those with CBD (Figs 2A and 4A). Tube feeding (*P* *=* 0.006) and a bedridden state (*P* *=* 0.002) appeared earlier in patients with CBD than in those with CBD mimics (Supplementary Fig. 3A and B). The interval from the initial symptoms to cognitive impairment, walking with assistance and the survival period were not significantly different between the two groups.

### CBS

#### Background pathology of CBS

Background pathologies of CBS (*n* = 48) included CBD (*n* = 16, 33.3%), followed by PSP (*n* = 14, 29.2%) and AD (*n* = 6, 12.5%; Fig. 1A and B).

### Comparison analysis among CBD–CBS, PSP–CBS, and AD–CBS

#### Initial symptoms and signs

We compared the initial symptoms of CBD–CBS with those of PSP–CBS and AD–CBS. The frequencies of frozen gait [*P*  *=* 0.005, OR (95% CI): 31.67 (1.46–685.34)] and short-step gait [*P* = 0.024, OR (95% CI): 15.75 (1.42–174.25)] were higher in the CBD–CBS group at onset than in the PSP–CBS group (Table 3). However, a distinct initial symptom of AD–CBS was not detected by the comparative analysis.

**Table 3:** Demographic data and initial signs in patients with CBD–CBS, PSP–CBS and AD–CBS

Feature	CBD–CBS (*n* = 16)	PSP–CBS (*n* = 14)	AD–CBS (*n* = 6)	*P*-value, OR [(95% CI); CBD–CBS versus PSP–CBS]	*P*-value, OR [(95% CI); CBD–CBS versus AD–CBS]	*P*-value, OR [(95% CI); PSP–CBS versus AD–CBS]
Age at onset, years	63.5 ± 8.5 (45–79)	67.8 ± 6.3 (59–79)	69.5 ± 11.6 (55–86)	0.132 (−9.95 to 1.38)	0.196 (−3.36 to 15.36)	0.670 (−10.02 to 6.59)
Age at death, years	71.6 ± 8.7 (50–86)	75.9 ± 5.9 (68–88)	77.8 ± 12.0 (62–93)	0.131 (−9.97 to 1.36)	0.195 (−3.45 to 15.87)	0.724 (−14.52 to 10.71)
Duration of disease, years	8.4 ± 3.4 (4–17)	8.6 ± 3.6 (4–18)	8.5 ± 1.8 (7–11)	0.873 (−2.81 to 2.40)	0.966 (−2.98 to 3.10)	0.928 (−3.12 to 3.41)
Gait disturbance	13/15 (87)	10/14 (71)	5/6 (83)	0.390, 2.60 (0.39–17.16)	1.000, 1.30 (0.10–17.73)	1.000,0.50 (0.04–5.74)
Slow gait	8/10 (80)	4/11 (36)	2/3 (67)	0.081, 7.00 (0.97–50.57)	1.000, 2.00 (0.11–34.82)	0.539, 0.29 (0.02–4.24)
Unstable gait	7/11 (64)	7/12 (58)	2/5 (40)	1.000, 1.25 (0.23–6.71)	0.596, 2.63 (0.30–22.00)	0.620, 2.10 (0.25–17.59)
Frozen gait	7/11 (64)	0/9 (0)	0/3 (0)	0.005*, 31.67 (1.46–685.34)	0.192, 11.67 (0.48–282.06)	1.000, 0.37 (0.01–22.39)
Short steps gait	7/11 (64)	1/10 (10)	3/5 (60)	0.024*, 15.75 (1.42–174.25)	1.000, 1.17 (0.13–10.22)	0.077, 0.67 (0.00–1.14)
Bradykinesia	8/11 (73)	8/12 (67)	3/4 (75)	1.000, 1.33 (0.22–7.98)	1.000, 0.89 (0.06–12.25)	1.000,0.67 (0.05–8.64)
Clumsy limbs	8/13 (62)	10/14 (71)	4/6 (67)	0.695, 0.64 (0.13–3.20)	1.000, 0.80 (0.10–6.10)	1.000, 1.25 (0.16–9.77)
Falls	5/11 (45)	5/13 (38)	1/5 (20)	1.000, 1.33 (0.26–6.81)	0.588, 3.33 (0.28–40.29)	0.615, 2.50 (0.21–29.26)
Amnesia	4/13 (31)	4/14 (29)	3/6 (50)	1.000, 1.11 (0.21–5.80)	0.617, 0.44 (0.06–3.24)	0.613, 0.40 (0.06–2.89)
Tremor	6/15 (40)	0/14 (0)	2/6 (33)	0.017*, 19.84 (0.00–394.8)	1.000, 1.33 (0.18–9.73)	0.079, 0.06 (0.00–1.51)
Speech disturbance	5/12 (42)	7/13 (54)	2/5 (40)	0.695, 0.61 (0.13–2.98)	1.000, 1.07 (0.13–8.98)	1.000, 1.75 (0.22–14.22)
Dysarthria	3/10 (30)	6/13 (46)	0/5 (0)	0.669, 0.50 (0.09–2.84)	0.506, 5.13 (0.22–121.11)	0.114, 9.53 (0.44–207.38)
Aphasia	1/10 (10)	3/13 (23)	0/4 (0)	0.604, 0.37 (0.034.23)	1.000, 1.42 (0.05–42.22)	0.541, 3.00 (0.13–70.88)
Personality change	3/11 (27)	1/12 (8)	1/4 (25)	0.317, 4.13 (0.36–47.31)	1.000, 1.13 (0.08–15.51)	0.450, 0.27 (0.01–5.77)
Lack of insight	3/12 (25)	1/10 (10)	1/3 (33)	0.594, 3.00 (0.26–34.58)	1.000, 0.67 (0.04–10.25)	0.423, 0.22 (0.01–5.28)
Apraxia	3/11 (27)	6/12 (50)	4/5 (80)	0.400, 0.38 (0.07–2.14)	0.106, 0.09 (0.01–1.21)	0.338, 0.25 (002–2.94)
Behavioural change	1/13 (8)	1/12 (8)	0/5 (0)	1.000, 0.92 (0.05–16.50)	1.000, 1.32 (0.05–37.78)	1.000, 1.43 (0.05–41.23)
Irritability	1/13 (8)	2/11 (18)	1/4 (25)	0.576, 0.38 (0.03–4.81)	0.427, 0.25 (0.01–5.26)	1.000, 0.67 (0.04–10.25)

Data are presented as mean ± SD (range) or *n* (%). Bold number indicates statistical significance. AD, Alzheimer's disease; CBD, corticobasal degeneration; CBS, corticobasal syndrome; PSP, progressive supranuclear palsy [*P < 0.05 (*t*-test or Fisher’s exact test)]; OR, odds ratio; 95% CI, 95% confidence interval.

#### Clinical symptoms and signs

The prevalence of dysarthria was higher in the PSP–CBS group at presentation than in the CBD–CBS group [*P* = 0.047, OR (95% CI): 6.75 (1.16–39.20)], and the frequency of supranuclear gaze palsy was higher in the PSP–CBS group during the entire course than in the CBD–CBS group [*P* = 0.030, OR (95% CI): 14.00 (1.39–141.49)]. In contrast, the frequency of pyramidal signs was higher in AD–CBS patients at presentation than in CBD–CBS patients [*P* = 0.012, OR (95% CI): 24.82 (1.17–527.15); Tables 4 and 5].

**Table 4:** Frequency of clinical features at presentation in patients with CBD–CBS, PSP–CBS and AD–CBS

Features at presentation	CBD–CBS (*n* = 16)	PSP–CBS (*n* = 14)	AD–CBS (*n* = 6)	*P*-value, OR [(95% CI); CBD–CBS versus PSP–CBS]	*P*-value, OR [(95% CI); CBD–CBS versus AD–CBS]	*P*-value, OR [(95% CI); PSP–CBS versus AD–CBS]
Limb rigidity or bradykinesia	15/15 (100)	12/14 (86)	5/6 (83)	0.224, 6.20 (0.27–144.32)	0.286, 8.45 (0.30–239.83)	1.000, 1.20 (0.09–16.44)
Limb rigidity or bradykinesia with asymmetric presentation	11/15 (73)	11/14 (79)	4/6 (67)	1.000, 0.75 (0.14–4.17)	1.000, 1.38 (0.18–10.65)	0.613, 1.83 (0.22–15.33)
Gait disturbance	12/15 (80)	10/12 (83)	5/6 (83)	1.000, 0.80 (0.11–5.77)	1.000, 0.80 (0.07–9.67)	1.000, 1.00 (0.07–13.87)
Slow gait	8/12 (67)	6/12 (50)	2/3 (67)	0.680, 2.00 (0.38–10.41)	0.593, 1.00 (0.07–14.64)	1.000, 0.50 (0.04–7.10)
Unstable gait	7/13 (54)	9/12 (75)	3/6 (50)	0.411, 0.39 (0.07–2.13)	1.000, 1.17 (0.17–8.09)	0.344, 3.00 (0.38–23.68)
Frozen gait	6/12 (50)	2/12 (17)	0/4 (0)	0.193, 5.00 (0.75–33.21)	0.234, 9.00 (0.40–203.31)	1.000, 2.14 (0.08–54.23)
Short-step gait	6/11 (55)	2/12 (17)	3/5 (60)	0.089, 6.00 (0.87–41.22)	1.000, 0.80 (0.09–6.85)	0.117, 0.13 (0.01–1.39)
Postural instability or falls	9/16 (56)	7/12 (58)	1/4 (25)	1.000, 0.92 (0.20–4.17)	0.582, 3.86 (0.33–45.57)	0.569, 4.20 (0.33–53.13)
Dystonia	8/14 (57)	6/12 (50)	0/4 (0)	1.000, 1.33 (0.28–6.28)	0.092, 11.77 (0.53–259.98)	0.234, 9.00 (0.40–203.31)
Dystonia with asymmetric presentation	5/14 (36)	6/12 (50)	0/4 (0)	0.692, 0.56 (0.12–2.68)	0.278, 5.21 (0.23–116.22)	0.234, 9.00 (0.40–203.31)
Tremor	3/14 (21)	2/13 (15)	1/5 (20)	1.000, 1.50 (0.21–10.81)	1.000, 1.09 (0.09–13.78)	1.000, 0.73 (0.05–10.39)
Myoclonus	4/15 (27)	2/13 (15)	2/5 (40)	0.655, 2.00 (0.30–13.27)	0.613, 0.55 (0.07–4.56)	0.533, 0.27 (0.03–2.83)
Myoclonus with asymmetric presentation	3/15 (20)	2/13 (15)	1/5 (20)	1.000, 1.38 (0.19–9.83)	1.000, 1.00 (0.08–12.56)	1.000, 0.73 (0.05–10.39)
Cerebellar ataxia	1/14 (7)	0/13 (0)	0/6 (0)	1.000, 3.00 (0.11–80.40)	1.000, 1.44 (0.05–40.54)	1.000, 0.48 (0.01–27.09)
Cognitive impairment (general)	8/14 (57)	9/14 (64)	2/6 (33)	1.000, 0.74 (0.16–3.39)	0.629, 2.67 (0.36–19.71)	0.336, 3.60 (0.48–27.11)
Executive dysfunction	8/12 (67)	8/10 (80)	1/2 (50)	0.644, 0.50 (0.07–3.55)	1.000, 2.00 (0.10–41.01)	0.455, 4.00 (0.17–95.76)
Behavioural changes	3/14 (21)	1/11 (9)	1/4 (25)	0.604, 2.73 (0.24–30.67)	1.000, 0.872 (0.06–10.00)	0.476, 0.30 (0.01–6.38)
Personality change	2/12 (17)	1/12 (8)	3/6 (50)	1.000, 2.20 (0.17–28.14)	0.268, 0.20 (0.02–1.02)	0.083, 0.09 (0.01–1.22)
Limb apraxia	6/12 (50)	8/13 (62)	3/4 (75)	0.695, 0.63 (0.13–3.07)	0.585, 0.33 (0.03–4.19)	1.000, 0.53 (0.04–6.66)
Cortical sensory	3/10 (30)	3/7 (43)	0/2 (0)	0.644, 0.57 (0.08–4.30)	1.000, 2.33 (0.09–62.69)	0.500, 3.89 (0.14–109.00)
Cortical sensory with asymmetric presentation	3/10 (30)	3/7 (43)	0/2 (0)	0.644, 0.57 (0.08–4.30)	1.000, 2.33 (0.09–62.69)	0.500, 3.89 (0.14–109.00)
Alien limb	0/13 (0)	2/11 (18)	0/2 (0)	0.199, 0.14 (0.01–1.98)	1.000, 0.19 (0.00–11.70)	1.000, 1.32 (0.05–37.16)
Alien limb with asymmetric presentation	0/13 (0)	0/11 (0)	0/2 (0)	1.000, 0.85 (0.02–46.42)	1.000, 0.22 (0.00–11.70)	1.000, 0.22 (0.00–13.81)
Groping, distorted speech production	4/13 (31)	3/10 (30)	1/4 (25)	1.000, 1.04 (0.17–6.23)	1.000, 1.33 (0.10–17.10)	1.000, 1.29 (0.09–17.96)
Supranuclear vertical gaze palsy or decreased velocity of vertical saccades	5/13 (38)	7/13 (54)	1/6 (17)	0.695, 0.54 (0.11–2.55)	0.605, 3.13 (0.28–35–16)	0.177, 5.83 (0.52–64.83)
Urinary incontinence	7/15 (47)	4/12 (33)	1/4 (25)	0.696, 1.75 (0.36–8.42)	0.603, 2.63 (0.22–31.35)	1.000, 1.50 (0.12–19.44)
Speech and language impairment	7/13 (54)	7/11 (64)	4/5 (80)	0.697, 0.67 (0.13–3.45)	0.596, 0.29 (0.03–3.37)	1.000, 0.44 (0.04–5.40)
Dysarthria	4/13 (31)	9/12 (82)	0/4 (0)	0.047*, 6.75 (1.16–39.20)**	0.519, 4.26 (0.19–97.49)	0.019*, 24.43 (1.03–580.66)
Slurred speech	2/14 (14)	3/12 (25)	0/2 (0)	0.635, 0.50 (0.07–3.65)	1.000, 1.00 (0.04–27.83)	1.000, 1.84 (0.07–48.68)
Pyramidal sign	5/15 (33)	4/13 (31)	6/6 (100)	1.000, 1.13 (0.23–5.54)	0.012*, 24.82 (1.17–527.15)***	0.011*, 27.44 (1.25–601.61)****

Data are presented as *n* (%). Bold number indicates statistical significance. AD, Alzheimer's disease; CBD, corticobasal degeneration; CBS, corticobasal syndrome; PSP, progressive supranuclear palsy [*P < 0.05 (Fisher’s exact test)]; OR, odds ratio; 95% CI, 95% confidence interval; OR (95% CI)**, OR [(95% CI); PSP–CBS versus CBD–CBS]; OR (95% CI)***, OR [(95% CI); AD–CBS versus CBD–CBS)]; OR (95% CI)****, OR [(95% CI); AD–CBS versus PSP–CBS].

**Table 5:** Frequency of clinical feature during the entire course in patients with CBD–CBS, PSP–CBS and AD–CBS

Features during the entire course	CBD–CBS (*n* = 16)	PSP–CBS (*n* = 14)	AD–CBS (*n* = 6)	*P*-value, OR [(95% CI); CBD–CBS versus PSP–CBS]	*P*-value, OR [(95% CI); CBD–CBS versus AD–CBS]	*P*-value, OR [(95% CI); PSP–CBS versus AD–CBS]
Limb rigidity or bradykinesia	15/15 (100)	14/14 (100)	5/5 (100)	1.000, 1.07 (0.02–57.49)	1.000, 2.82 (0.05–159.97)	1.000, 2.64 (0.05–149.97)
Limb rigidity or bradykinesia with asymmetric presentation	11/15 (73)	13/14 (93)	4/5 (80)	0.330, 0.21 (0.02–2.18)	1.000, 0.69 (0.06–8.15)	0.468, 3.25 (0.10–64.62)
Gait disturbance	16/16 (100)	14/14 (100)	5/6 (83)	1.000, 1.14 (0.02–61.08)	0.273, 9.00 (0.32–254.73)	0.300, 7.91 (0.28–224.93)
Slow gait	12/12 (100)	11/13 (85)	2/3 (67)	0.480, 5.43 (0.24–125.60)	0.200, 15.00 (0.46–485.35)	0.489, 2.75 (0.16–46.79)
Unstable gait	14/14 (100)	12/12 (100)	5/6 (83)	1.000, 1.16 (0.02–62.85)	0.300, 7.91 (0.28–224–93)	0.333, 6.82 (0.24–195.14)
Frozen gait	8/13 (62)	4/10 (40)	1/3 (33)	0.414, 2.40 (0.44–12.98)	0.550, 3.20 (0.23–45.19)	1.000, 1.33 (0.09–20.11)
Short-step gait	10/12 (83)	6/12 (50)	3/5 (60)	0.193, 5.00 (0.75–33.21)	0.538, 3.33 (0.32–34.83)	1.000, 0.67 (0.00–3.94)
Postural instability or falls	15/16 (94)	12/13 (92)	3/4 (75)	1.000, 1.25 (0.07–22.131)	0.368, 5.00 (0.24–104.15)	0.427, 4.00 (0.19–84.20)
Dystonia	10/13 (77)	8/12 (67)	0/2 (0)	0.673, 1.67 (0.29–9.71)	0.095, 15.00 (0.57–394.09)	0.165, 9.44 (0.37–242.19)
Dystonia with asymmetric presentation	7/13 (54)	8/12 (67)	0/2 (0)	0.688, 0.58 (0.12–2.95)	0.467, 5.77 (0.23–143.38)	0.165, 9.44 (0.37–242.19)
Tremor	5/15 (33)	2/13 (15)	1/5 (20)	0.396, 2.75 (0.43–17.49)	1.000, 2.00 (0.17–22.95)	1.000, 0.73 (0.05–10.39)
Myoclonus	6/14 (43)	2/12 (17)	5/6 (83)	0.216, 3.75 (0.59–23.87)	0.157, 0.15 (0.01–1.64)	0.013*, 25.00 (1.80–346.71)***
Myoclonus with asymmetric presentation	4/14 (29)	2/12 (17)	3/6 (50)	0.652, 2.00 (0.30–13.51)	0.613, 0.40 (0.06–2.89)	0.268, 0.20 (0.02–1.82)
Cerebellar ataxia	1/13 (8)	2/12 (17)	0/4 (0)	0.593, 0.42 (0.03–5.30)	1.000, 1.08 (0.04–31.63)	1.000, 2.14 (0.08–54.23)
Cognitive impairment (general)	12/14 (86)	11/12 (92)	5/5 (100)	1.000, 0.55 (0.04–6.89)	1.000, 0.45 (0.02–11.13)	1.000, 0.70 (0.02–20.03)
Executive dysfunction	8/10 (80)	8/8 (100)	3/3 (100)	0.477, 0.20 (0.01–4.82)	1.000, 0.49 (0.02–12.93)	1.000, 2.43 (0.04–148.44)
Behavioural changes	7/13 (54)	2/11 (18)	2/3 (67)	0.105, 5.25 (0.80–34.43)	1.000, 0.58 (0.04–8.15)	0.176, 0.11 (0.01–1.92)
Personality change	3/11 (27)	1/11 (9)	4/5 (80)	0.587, 3.75 (0.32–43.31)	0.106, 0.09 (0.01–1.21)	0.013*, 40.00 (1.98–807.14)***
Limb apraxia	8/10 (80)	10/13 (77)	4/4 (100)	1.000, 1.20 (0.16–9.01)	1.000, 0.38 (0.01–9.69)	0.541, 0.33 (0.01–7.88)
Cortical sensory	4/11 (36)	4/7 (57)	0/2 (0)	0.631, 0.43 (0.06–2.97)	1.000, 3.00 (0.12–77.65)	0.444, 6.43 (0.23–181.83)
Cortical sensory with asymmetric presentation	4/11 (36)	4/7 (57)	0/2 (0)	0.631, 0.43 (0.06–2.97)	1.000, 3.00 (0.12–77.65)	0.444, 6.43 (0.23–181.83)
Alien limb	2/13 (15)	4/11 (36)	0/1 (0)	0.357, 0.32 (0.05–2.22)	1.000, 0.65 (0.02–21.18)	1.000, 1.80 (0.06–54.33)
Alien limb with asymmetric presentation	0/13 (0)	0/11 (0)	0/1 (0)	1.000, 0.85 (0.02–46.42)	1.000, 0.11 (0.00–7.93)	1.000, 0.13 (0.00–9.36)
Groping, distorted speech production	7/8 (88)	4/9 (44)	2/3 (67)	0.131, 8.75 (0.24–103.83)	0.491, 3.50 (0.14–84.70)	1.000, 0.40 (0.03–6.18)
Supranuclear vertical gaze palsy or decreased velocity of vertical saccades	6/13 (46)	12/13 (92)	4/6 (67)	0.030*, 14.00 (1.39–141.49)**	0.629, 0.43 (0.06–3.22)	0.222, 6.00 (0.42–85.25)
Urinary incontinence	11/13 (85)	8/12 (67)	3/3 (100)	0.378, 2.75 (0.40–18.88)	1.000, 0.66 (0.03–17.18)	0.517, 0.27 (0.01–6.46)
Speech and language impairment	12/13 (92)	10/13 (77)	5/5 (100)	0.593, 3.60 (0.32–40.23)	1.000, 0.76 (0.13–21.68)	0.522, 0.27 (0.01–6.29)
Dysarthria	10/13 (77)	10/11 (83)	2/3 (67)	0.596, 0.33 (0.03–3.78)	1.000, 1.67 (0.11–25.43)	0.396, 5.00(0.21–117.90)
Slurred speech	4/11 (36)	3/10 (30)	0/1 (0)	1.000, 1.33 (0.21–8.29)	1.000, 1.80 (0.06–54.33)	1.000, 1.40 (0.04–43.79)
Pyramidal sign	7/13 (54)	6/13 (46)	6/6 (100)	0.717, 1.36 (0.29–6.36)	0.109, 0.09 (0.00–1.90)	0.044*, 0.07 (0.00–1.43)

Data are presented as *n* (%). Bold number indicates statistical significance. AD, Alzheimer's disease; CBD, corticobasal degeneration; CBS, corticobasal syndrome; PSP, progressive supranuclear palsy [*P < 0.05 (Fisher’s exact test)]; OR, odds ratio; 95% CI, 95% confidence interval; OR (95% CI)**, OR [(95% CI); PSP–CBS versus CBD–CBS]; OR (95% CI)***, OR [(95% CI); AD–CBS versus PSP–CBS].

The frequency of dysarthria was higher in patients with PSP–CBS than in those with AD–CBS [*P*  *=* 0.019, OR (95% CI): 24.43 (1.03–580.46)] at presentation. In contrast, the frequency of pyramidal signs at presentation [*P* = 0.011, OR (95% CI): 27.44 (1.25–601.61)] was higher in patients with ADe–CBS (Table 4). The frequencies of myoclonus [*P*  *=* 0.013, OR (95% CI): 25.00 (1.80–346.71)] and personality change [*P*  *=* 0.013, OR (95% CI): 40.00 (1.98–807.14)] were higher in patients with AD–CBS during the entire course (Table 5).

### Findings suggestive of background pathology from the decision tree analysis

**Figure 5:**
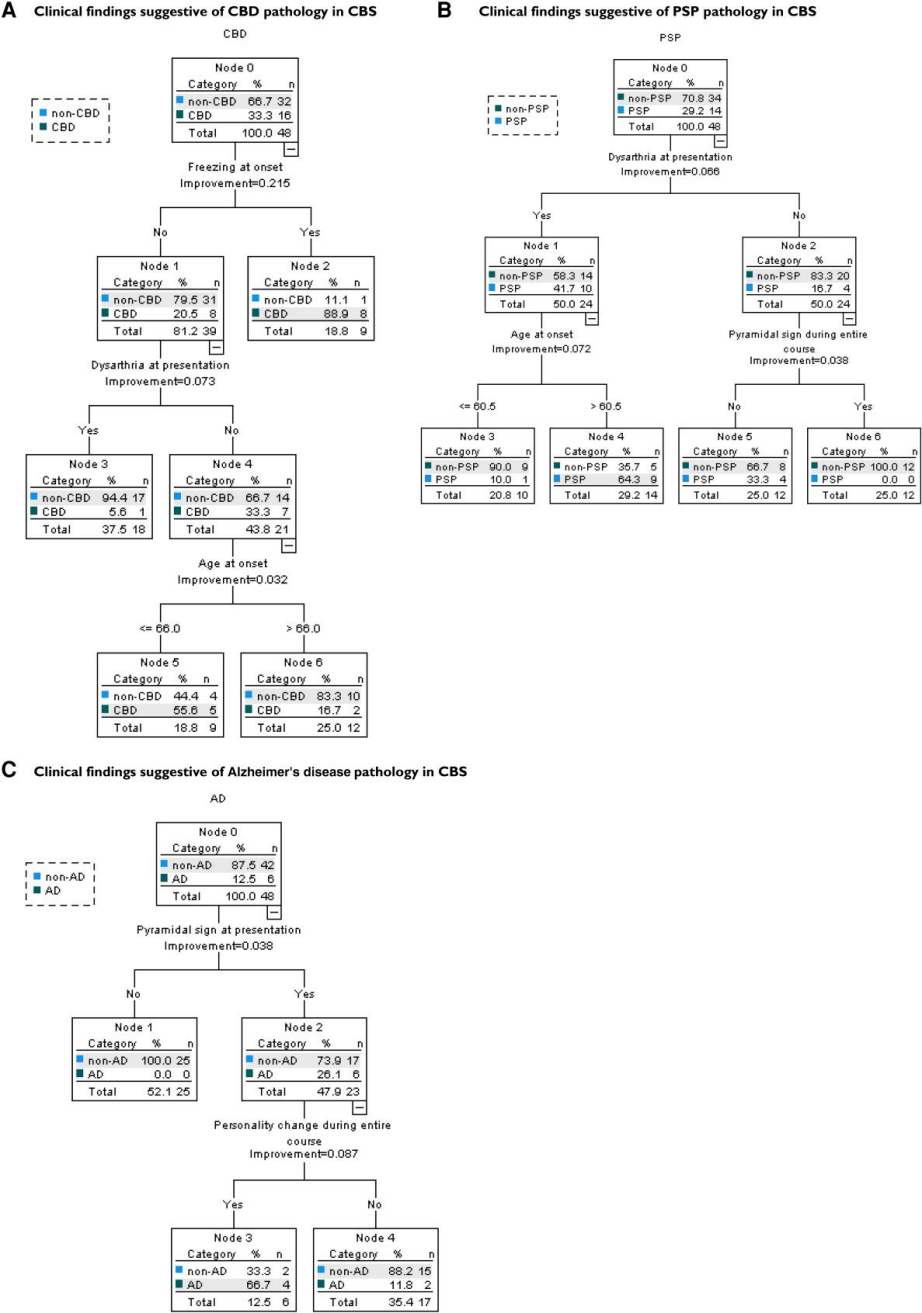
Decision tree analysis for background pathology of CBS. A decision tree analysis was performed using the classification and regression tree method with CBD, PSP and AD as the dependent variables and sex, age at onset and significant findings (freezing at onset, tremor at onset, dysarthria at onset, dysarthria at presentation, pyramidal signs at presentation, pyramidal signs during the entire course, myoclonus during the entire course, personality change during the entire course and supranuclear gaze palsy during the entire course) as the independent variables, and cross-validation was performed. (**A**) Clinical findings suggestive of CBD pathology. ‘Freezing at onset’ or ‘no dysarthria at presentation and age at onset <66 years in the case without freezing at onset’ predicted CBD pathology with a sensitivity of 81.3%, specificity of 84.4%, PPV of 72.3% and NPV of 90%. (**B**) Clinical findings suggestive of PSP pathology. ‘Dysarthria at presentation and age at onset older than 61 years’ suggested PSP pathology, with a sensitivity of 64.3%, specificity of 85.3%, PPV of 64.3% and NPV of 85.3%. (**C**) Clinical findings suggestive of AD pathology. ‘Pyramidal sign at presentation and personality change during the entire course’ implied AD pathology with a sensitivity of 66.7%, specificity of 95.2%, PPV of 66.7% and NPV of 95.2%. n, number of patients; CRT, classification and regression tree method; CBS, corticobasal syndrome; CBD, corticobasal degeneration; PSP, progressive supranuclear palsy; AD, Alzheimer's disease; PPV, positive predictive value; NPV, negative predictive value.

Decision tree analysis in CBS showed that ‘freezing at onset’ or ‘no dysarthria at presentation and age at onset <66 years in the case without freezing at onset’ predicted CBD pathology with a sensitivity of 81.3% (13/16) and specificity of 84.4% (27/32), ‘dysarthria at presentation and age at onset older than 61 years’ suggested PSP pathology with a sensitivity of 64.3% (9/14) and specificity of 85.3% (29/34) and ‘pyramidal sign at presentation and personality change during the entire course’ implied AD pathology with a sensitivity of 66.7% (4/6) and specificity of 95.2% (40/42; Fig. 5).

### Interval from the initial symptoms to a key milestone

We compared the intervals from the initial symptoms to the key milestones between CBD–CBS and PSP–CBS or AD–CBS. Within the early 2 years, patients with CBD–CBS initially presented with gait disturbance (median 0.0 years), followed by falls (1.5 years; Fig. 2B). However, those with PSP–CBS initially presented behaviour changes and gait disturbance (0.0 year), followed by speech impairment and falls (1.0 years), and later accompanied supranuclear gaze palsy (6.0 years; Fig. 4B). Patients with AD–CBS initially presented with gait disturbance (0.0 years), followed by cognitive impairment (1.0 years), speech impairment and falls (2.0 years; Fig. 4C).

## Discussion

### Clinical characteristics of patients with CBD pathology

The present study is the first to show the clinical spectrum and course of the genetic, biochemical and pathological confirmation of CBD in Japan.

### Age at onset and death

In the present study, the mean age at onset of CBD was in the mid-60s, similar to previous studies.^8–13,48^ Although an age at onset of ≥50 years is required for probable sporadic CBD of Armstrong’s criteria, one patient with CBD (Patient no. 21) developed symptoms at 45 years of age, the same as the youngest age reported previously.^48^ In previous studies, a patient with an onset at age 83 (Patient no. 11) had the oldest onset.^8–14,48^ Age at death was consistent with the published data.^8–14,48^ The ratio of men and women was equal, similar to those in western countries.^8–14,48^

### Initial signs and symptoms

In terms of initial signs and symptoms, many studies reported that limb clumsiness was the most common initial symptom (37–50%).^12,48^ Other signs at onset reported were gait disorder,^12,48^ falls,^48^ sensory problems,^12,48^ behavioural change,^12,48^ cognitive problems (memory loss)^12,48^ and tremor.^48^ Although limb clumsiness was also seen as often as in previous studies (48%), the most common finding at onset was gait disturbance (74%), which was more frequent than in previous reports.^9,12^ This may have been associated with the high proportion of participants from the department of neurology in our cohort. The most frequent gait characteristic at onset was slow gait (57%), followed by unstable gait (48%). Surprisingly, the prevalence of gait freezing was noted in ∼40% of the patients.

### Clinical signs and symptoms

Almost all patients with CBD developed limb rigidity or bradykinesia, gait disturbances and postural instability. These motor signs were more frequent than those reported by Armstrong *et al.*^9^ Characteristics of gait (e.g. short stepped, bradykinetic, unstable and broad based) were the same as those in previous studies.^12,48^ In PSP, gait freezing is the primary sign of akinesia, especially as patients with PSP–PGF develop gait freezing in their early stages.^43^ Gait freezing was also found for the first time in CBD, which has the same 4R tauopathies. Both dystonia and myoclonus were the major signs of CBS, but they were less frequent, which was consistent with previous studies.^8,9,12,14^ The cognitive impairment seen in 90% of patients with CBD was the most common higher cortical feature, as previously described.^8,9,13,14^ More than 80% of the patients with CBD presented executive dysfunction; it was more frequent than in previous reports.^12^ Behavioural changes were noted in 56% of patients with CBD during the entire course, as frequently as Armstrong *et al*.^9^ Patients who developed apraxia were 29% at presentation and 48% during the entire course, which was less than in previous studies.^9,14^ As previous reports^9,14^ pointed out, cortical sensory loss (21%) and alien limb phenomena (7%) were infrequent despite the core features of CBS. Therefore, few patients with CBD met possible (46%) or probable (4%) CBS as per Armstrong *et al.*’s^9^ criteria (Table 2). However, the proportion of cortical sensory loss evaluated was particularly small (only 20 of 32 patients at presentation and 19 during the entire course), and it is possible that the actual number might be much higher, and the proportion of CBS may also be higher.

### Clinical phenotype and course

In the present study, the frequencies of CBS and naPPA were less than those reported by Alexander *et al*.^14^ FBS tended to have the same frequency, and the proportion of PSPS tended to be higher than that reported by Alexander *et al*.^14^ This difference may be because many patients came from movement disorders and not from dementia in this cohort. Many patients also had more than one clinical type (Fig. 3). In addition, half of the patients with CBD pathology developed non-CBS phenotypes such as PSP and dementia, and their physicians did not suspect CBD pathology, thereby not formulating an evaluation of the primary symptoms of CBS. Evaluation throughout the course of the disease was particularly difficult, in part because the patients eventually becoming bedridden and mutic, therefore making the evaluation for aphasia or speech incomplete.

Kertesz *et al*.^49^ reported in detail the time course of the clinical type of 12 pathologically confirmed CBD patients in a prospective cohort of FTD. The most common first clinical syndrome was primary progressive aphasia, followed by behavioural variant of FTD, and CBD syndrome. The clinical phenotype evolved to the other second and third syndromes over time. As mentioned above, it has been shown that clinical syndrome evolves over time in CBD. To our knowledge, this study is the first to describe the onset of each clinical sign and symptom by clinical phenotype in patients with pathologically confirmed CBD. In CBD, the earliest symptom was gait disturbance, followed by behavioural changes (median 1.0 year), falls and cognitive impairment (2.0 years, respectively; Fig. 2A). The clinical course differs depending on the clinical type (Fig. 2B–D).

### Survival and cause of death

Some studies reported that the early presence of parkinsonism,^48^ frontal lobe features^48^ and dementia^12^ predicted shorter survival^48^ in pathologically confirmed CBD. Age at onset was not associated with survival, consistent with previous studies.^12,48^ No previous reports have investigated whether the basic clinical characteristic of sex affects survival; our findings showed that survival was predicted to be longer in women than it was in men.

In patients with CBD, the most common cause of death was pneumonia, similar to the findings of previous studies.^48^

### Background pathology of CBS

In the present study, the background pathology of CBS in Japan constituted a variety of proteinopathies: CBD was the major (33.3%), followed by PSP (29.2%) and AD (12.5%). In previous reports on the background pathology of CBS, PSP and AD were found to be the two major diseases (other than CBD). PSP was observed in 0.0–47.6% of CBS,^7,10,11,13,18–20,26,27,50^ while AD was observed in 0.0–50.0% of CBS.^7,10,11,13,16,18–20,26,27,50^ Generally, the frequency of PSP was higher in cohorts who were treated mainly for movement disorders than those treated mainly for cognitive impairment.^7,10,11^ On the contrary, the frequency of AD was higher in cohorts than those treated mainly for cognitive impairment.^19,27^ The result of our cohort from treating mainly movement disorders is similar to the previous cohorts that mainly aimed to treat movement disorders, as well.

### Clinical characteristics of CBD–CBS, PSP–CBS and AD–CBS

Finally, we discuss the clinical characteristics of each disease type. Gait disturbance at onset and early falls were common signs in CBD–CBS, PSP–CBS and Alzheimer’s disease–CBS.

### CBD–CBS

The clinical characteristics of CBD–CBS are gait disturbances initially, followed by falls within 2 years and later accompanied by behavioural changes and cognitive impairment. At the onset, CBD–CBS patients more frequently presented with frozen gait, and short-step gait, compared with PSP–CBS; the frequency of short-step gait in CBD–CBS was almost equal to that in AD–CBS. Frozen gait has not been previously reported in CBD. As per our study, frozen gait may suggest CBD–CBS.

### PSP–CBS

Both PSP and CBD are 4R tauopathies having overlapping clinical phenotypes; with CBS having the PSP phenotype and PSPS having the clinical phenotype of CBD, distinguishing them becomes extremely difficult clinically. However, our analysis revealed some differences between the two. The clinical characteristics of PSP–CBS include gait disturbance at onset, followed by speech disturbance and falls within 2 years and later the accompaniment of supranuclear gaze palsy. Supranuclear gaze palsy was more frequently observed during the course, compared with CBD–CBS. Supranuclear gaze palsy is a late sign, as per Respondek *et al*.,^51^ but falling down appeared earlier (1.0 year) in PSP–CBS. Moreover, patients with PSP–CBS more frequently presented with dysarthria at presentation than with CBD–CBS or AD–CBS. Although dysarthria has not been previously reported as an early symptom, the existence of dysarthria at presentation may be suggestive of PSP–CBS.

### AD–CBS

The clinical characteristics of AD–CBS are initial gait disturbance, followed by cognitive impairment. Shelly *et al*.^27^ previously reported that asymmetric extrapyramidal signs were observed, and cognitive impairment was an earlier symptom of AD–CBS than CBD–CBS; however, the previous report did not compare it with PSP–CBS. In our cohort, a higher frequency of myoclonus was observed at diagnosis and during the entire course of AD–CBS than that in PSP–CBS. Hu *et al*.^37^ previously reported that a higher frequency of myoclonus was observed in AD–CBS than in CBD–CBS but not in PSP–CBS. In addition, in our cohort, personality change and pyramidal signs were more frequently observed in patients with AD–CBS than in those with PSP–CBS. These findings may be related to hippocampal-sparing type AD in which the primary motor cortex has higher neurofibrillary tangle (NFT) counts than typical AD^52^

### Findings suggestive of background pathology from the decision tree analysis

The results of the decision tree analysis revealed that ‘the presence of frozen gait at onset’ or ‘the absence of dysarthria at presentation and younger onset’ suggests CBD pathology; ‘dysarthria at presentation and age at onset older than 61 years’ predicted PSP pathology; furthermore, ‘pyramidal sign at presentation and personality change during the entire course’ implied AD pathology. Although these findings have not been previously reported, they may contribute to a correct diagnosis during life. The sensitivity and specificity of the decision tree analysis for predicting background pathology are still not high, and if used alone, it will have limited diagnostic value.

### Limitation

This study had some limitations. Firstly, this was a small, retrospective cohort study. Furthermore, we could not completely elucidate the patients’ symptoms and signs throughout the clinical course and could obtain the items considered present or absent only if described. Therefore, in some analyses, the comparison between the two groups would have had an insufficient number of patients. Moreover, the patient’s information on the biomarkers to exclude AD was inadequate in the study. A limitation of the study is also that the lack of information (which is already mentioned) made it hard to assess the Armstrong’s CBD criteria. However, a strength of the study is that the findings could still inform future revisions of the criteria to improve sensitivity and specificity. Therefore, a large international multicentre prospective cohort study is required for further investigating CBD and CBS. This study focused on clinical features, and its results may be combined with highly predictive biomarkers to detect background pathologies.^53^

## Conclusion

To our knowledge, this study is the first to describe the clinical spectrum of the pathologically, genetically and biochemically verified patients with CBD. This is also the first report of the clinical differences between CBD–CBS, PSP–CBS and AD–CBS. In CBS, gait disturbance at onset and early falls are common signs. However, frozen gait at onset may suggest CBD pathology, dysarthria may predict PSP pathology and personality change and pyramidal signs may imply AD pathology.

## Supplementary Material

fcad296_Supplementary_Data

## Data Availability

Data supporting the findings of this study are available in the article and its Supplementary material. All supporting data are available from the corresponding authors upon request.

## Appendix

Collaborative group of the J-VAC study

Yuichi Hayashi, Takayoshi Shimohata, Mari Yoshida, Yuko Saito, Koichi Wakabayashi, Takashi Komori, Masato Hasegawa, Takeshi Ikeuchi, Aya M Tokumaru, Keita Sakurai, Shigeo Murayama, Kazuko Hasegawa, Toshiki Uchihara, Yasuko Toyoshima, Yufuko Saito, Ichiro Yabe, Satoshi Tanikawa, Keizo Sugaya, Kentaro Hayashi, Terunori Sano, Masaki Takao, Motoko Sakai, Harutoshi Fujimura, Hiroshi Takigawa, Tadashi Adachi, Ritsuko Hanajima, Osamu Yokota, Tomoko Miki, Yasushi Iwasaki, Michio Kobayashi, Nobutaka Arai, Takuya Ohkubo, Takanori Yokota, Keiko Mori, Masumi Ito, Chiho Ishida, Masaharu Tanaka, Jiro Idezuka, Masato Kanazawa, Kenju Aoki, Masashi Aoki, Takafumi Hasegawa, Hirohisa Watanabe, Atsushi Hashizume, Hisayoshi Niwa, Keizo Yasui, Keita Ito, Yukihiko Washimi, Eiichiro Mukai, Akatsuki Kubota, Tatsushi Toda, Kenji Nakashima, Shinya Tanaka, Kinya Ishikawa, Renpei Sengoku, Yasuhiro Sakashita, Tomoyasu Matsubara, Kimiko Inoue, Chiaki Mori, Tomoko Saito, Takahiko Tokuda, Hisanori Kowa, Seishi Terada, Hanae Nakashima-Yasuda, Yuko Kato-Motozaki, Kiyonobu Komai, Osamu Onodera, Akiyoshi Kakita, Hiroshi Shimizu, Mari Tada, Arifumi Matsumoto, Akio Kikuchi, Mutsufusa Watanabe, Masahisa Katsuno, Tosiaki Ieda, Meiko Hashimoto Maeda and Ikuko Aiba. Details are available in the Supplementary material.
