# Correction to: Clinical course of pathologically confirmed corticobasal degeneration and corticobasal syndrome

**DOI:** 10.1093/braincomms/fcae028

**Published:** 2024-02-12

This is a correction to: Ikuko Aiba, Yuichi Hayashi, Takayoshi Shimohata, Mari Yoshida, Yuko Saito, Koichi Wakabayashi, Takashi Komori, Masato Hasegawa, Takeshi Ikeuchi, Aya M Tokumaru, Keita Sakurai, Shigeo Murayama, Kazuko Hasegawa, Toshiki Uchihara, Yasuko Toyoshima, Yufuko Saito, Ichiro Yabe, Satoshi Tanikawa, Keizo Sugaya, Kentaro Hayashi, Terunori Sano, Masaki Takao, Motoko Sakai, Harutoshi Fujimura, Hiroshi Takigawa, Tadashi Adachi, Ritsuko Hanajima, Osamu Yokota, Tomoko Miki, Yasushi Iwasaki, Michio Kobayashi, Nobutaka Arai, Takuya Ohkubo, Takanori Yokota, Keiko Mori, Masumi Ito, Chiho Ishida, Masaharu Tanaka, Jiro Idezuka, Masato Kanazawa, Kenju Aoki, Masashi Aoki, Takafumi Hasegawa, Hirohisa Watanabe, Atsushi Hashizume, Hisayoshi Niwa, Keizo Yasui, Keita Ito, Yukihiko Washimi, Eiichiro Mukai, Akatsuki Kubota, Tatsushi Toda, Kenji Nakashima, J-VAC study group, Clinical course of pathologically confirmed corticobasal degeneration and corticobasal syndrome, *Brain Communications*, Volume 5, Issue 6, 2023, fcad296, https://doi.org/10.1093/braincomms/fcad296

Upon original publication, in the “Collaborative group of the J-VAC study” given in the Appendix and supplementary data of this manuscript, there was a typographical error in the name of Tomoyasu Matsubara.

This has been corrected.

