# Supplementary material for: Clinical course of pathologically confirmed corticobasal degeneration and corticobasal syndrome
Source: Brain Commun. 2023 Nov 3;5(6):fcad296. doi: 10.1093/braincomms/fcad296 (PMC10715783; doi:10.1093/braincomms/fcad296)
Supplement: fcad296_Supplementary_Data [file fcad296_supplementary_data.zip › Supplementary material.pdf]

Supplementary Table 1. Pathological diagnostic datasheet

Case ID	Neuronal loss & gliosis	Ballooned neurons	Tau- or Gallyas-positive neurons NFTs/pretangles	Tau- or Gallyas-positive astrocytic plaques	Tau- or Gallyas-positive coiled bodies	Tau- or Gallyas-positive thresds	Tau- or Gallyas-positive tufted astrocytes	Other Tau- or Gallyas-positive glial inclusions
**Cerebral cortex**								
Frontal								
Motor (peri-Rolandic)								
Cerebral white matter								
Parietal								
Temporal								
Entorhinal								
Occipital								
**Subcortical areas**								
Hippocampus								
Amygdala								
Basal nucleus of Meynert								
Caudate & putamen								
Globus pallidus								
Internal capsule								
Thalamus								
Subthalamic nucleus								
Midbrain tectum (colliculi)								
Red nucleus								
Substantia nigra								
Cerebral peduncle								
Locus coeruleus								
Pontine tegmentum								
Pontine nucleus								
Fibers in pontine base								
Inferior olivary nuclei								
**Cerebellum**								
Dentate nucleus								
Cerebellar white matter								
Final pathological CBD diagnosis	Yes	No	Pending					
Senile changes								
NFT Braak stage (Gallyas or AT8)	Stage 0	Stage I	Stage II	Stage III	Stage IV	Stage V	Stage VI	
	Other tauopathies	Not assessed						
CERAD neuritic plaques	No	Sparse	Moderate	Frequent	Not assessed			
NIA-AA Alzheimer's disease neuropathologic changes (ADNC)	Not AD	Low ADNC	Intermediate ADNC	High ADNC	Not assessed			
Argyrophilic grain	No	Saito's stage I	Saito's stage II	Saito's stage III	Not assessed			
Comments								

0=none, 1=minimal/mild, 2=moderate/severe, NA=not assessed

Supplementary Table 2. Pathological information of patients enrolled as having CBD pathology at each institution

J-VAC ID number	sex	Age at onset (y)	Age at death (y)	Disease duration (y)	Pathological diagnosis	NFT stage	Senile plaques Thal phase & CERAD	Argyrophilic grain, Saito's stage	Western blotting	*MAPT* mutation
1	m	66	75	9	PSP	II	0	0	not detected	-
2	f	63	78	15	CBD	II	1, 0	II	CBD pattern	-
3	f	59	65	6	CBD	II	1, 0	II	CBD pattern	-
4	m	61	67	6	CBD	II	1, 0	I	CBD pattern	-
5	m	41	58	17	minimum change	II	0	I	not detected	-
6	f	71	79	8	CBD	II	0	III	CBD pattern	-
7	f	74	84	10	CBD	II	0	I	CBD pattern	-
8	m	73	79	6	CBD	II	0	II	CBD pattern	-
9	f	68	74	6	CBD	II	0	II	CBD pattern	-
10	f	59	69	10	CBD	I	0	III	CBD pattern	-
11	f	83	87	4	CBD	II	3, B	III	CBD pattern	-
12	f	71	82	11	CBD	III	0	II	CBD pattern	-
13	m	65	68	3	CBD	II	0	0	CBD pattern	-
14	f	70	75	5	CBD	II	1, 0	I	not detected*	-
15[1]	m	79	86	7	CBD	II	2, A	III	CBD pattern	-
16	m	72	77	5	CBD	II	1, 0	II	CBD pattern	-
17	m	60	69	9	CBD	II	0	II	CBD pattern	-
18	m	53	60	7	CBD	I	0	II	CBD pattern	-
19	f	59	70	11	CBD	III	1, A	III	CBD pattern	-
20	f	63	71	8	CBD	I	1, 0	II	CBD pattern	-
21	m	45	50	5	CBD	II	NA	II	CBD pattern	-
22	m	56	60	4	CBD	II	NA	NA	CBD pattern	-
23	m	65	70	5	CBD	II	NA	NA	CBD pattern	-
24	m	58	67	9	CBD	I	0	NA	CBD pattern	-
25	m	70	76	6	PSP	II	NA	I	PSP pattern	-
26	f	75	84	9	CBD	II	0	I	CBD pattern	-
27	f	62	74	12	CBD	II	1, 0	II	CBD pattern	+
28	m	72	83	11	CBD	II	1, 0	III	CBD pattern	-
29	f	65	72	7	CBD	II	1, 0	I	CBD pattern	-
30	f	69	73	4	Atypical 4 repeat tau pathology, Lewy body pathology	II	2, B	I	CBD pattern	-
31	f	67	76	9	CBD	II	0	II	CBD pattern	-
32	m	66	70	4	CBD	II	3, B	I	CBD pattern	-
33[2]	f	65	73	8	CBD	III	1, 0	II	CBD pattern	-
34	m	64	70	6	CBD	II	0	II	CBD pattern	-
35	f	52	68	16	CBD	II	0	III	CBD pattern	-
36	m	72	76	4	CBD	II	1, 0	I	CBD pattern	-
37	m	72	78	6	CBD	III	0	I	CBD pattern	-

CBD: corticobasal degeneration; f: female; m: male; NA: not available; PSP: progressive supranuclear palsy; y: years or years old,
* concomitant bacterial meningitis in terminal stage.

Supplementary Table 3. Initial symptoms and signs in patients with CBD or CBD mimics

Feature	CBD<br>n = 32	CBD mimics<br>n = 32	P-value, OR [95% CI]<br>(CBD vs CBD mimics)
Gait disturbance	23 / 31 (74)	20 / 32 (69)	0.419, 1.72 [0.59-5.06]
Slow gait	13 / 23 (57)	9 / 24 (38)	0.248, 2.17 [0.67-6.96]
Unstable gait	11 / 23 (48)	13 / 28 (46)	1.000, 1.06 [0.35-3.19]
Frozen gait	9 / 23 (39)	0 / 22 (0)	**0.002*, 29.48 [1.59-546.35]**
Short steps gait	8 / 22 (36)	4 / 25 (16)	0.180, 3.00 [0.76-11.90]
Bradykinesia	16 / 25 (64)	14 / 26 (54)	0.573, 1.52 [0.50-4.69]
Clumsy limbs	13 / 27 (48)	19 / 30 (63)	0.293, 0.54 [0.19-1.55]
Falls	12 / 26 (46)	7 / 27 (26)	0.158, 2.45 [0.77-7.78]
Amnesia	13 / 29 (44)	12 / 30 (40)	0.795, 1.22 [0.43-3.43]
Tremor	11 / 29 (38)	3 / 31 (10)	**0.014*, 5.70 [1.40-23.30]**
Speech disturbance	10 / 28 (36)	16 / 29 (55)	0.187, 0.45 [0.16-1.31]
Dysarthria	5 / 22 (23)	12 / 29 (41)	0.233, 0.42 [0.12-1.44]
Aphasia	3 / 24 (13)	3 / 26 (12)	1.000, 1.10 [0.20-6.03]
Personality change	7 / 23 (30)	5 / 27 (19)	0.508, 1.93 [0.52-7.18]
Lack of insight	6 / 24 (25)	3 / 23 (13)	0.461, 2.22 [0.48-10.21]
Apraxia	6 / 25 (24)	2 / 27 (7)	0.134, 3.95 [0.72-21.78]
Behavioral change	6 / 27 (22)	2 / 28 (7)	0.143, 3.71 [0.68-20.34]
Irritability	4 / 26 (15)	4 / 26 (15)	1.000, 1.00 [0.22-4.51]
Hallucination	3 / 26 (12)	1 / 28 (4)	0.342, 3.52 [0.34-36.22]

Data are presented as n (%), CBD: corticobasal degeneration, *P < 0.05 (Fisher's exact test); OR; odds ratio; 95% CI: 95% confidence interval. **Bold number indicates statistically significant.**

Supplementary Table 4. Frequency of clinical features in patients with CBD or CBD mimics

Feature	CBD (n = 32)		CBD mimics (n = 32)		P-value, OR [95% CI] (CBD vs CBD mimics)	
	At presentation	During entire course	At presentation	During entire course	At presentation	During entire course
Limb rigidity or bradykinesia	27 / 31 (87)	28 / 31 (90)	27 / 32 (84)	31 / 31 (100)	1.000, 1.25 [0.30-5.17]	0.238, 0.13 [0.01-2.61]
with asymmetric presentation	*19 / 31 (61)*	*20 / 31 (65)*	*24 / 32 (75)*	*28 / 31 (90)*	*0.287, 0.53 [0.18-1.55]*	***0.031*, 5.13 [1.27-20.81]*****
Gait disturbance	24 / 30 (80)	30 / 31 (97)	23 / 30 (77)	31 / 32 (97)	1.000, 1.22 [0.36-4.17]	1.000, 0.97 [0.06-16.19]
Slow gait	16 / 26 (62)	21 / 23 (91)	12 / 25 (48)	23 / 26 (88)	0.404, 1.73 [0.57-5.28]	1.000, 1.37 [0.21-9.02]
Unstable gait	16 / 27 (59)	24 / 25 (96)	16 / 27 (59)	26 / 28 (93)	1.000, 1.00 [0.34-2.96]	1.000, 1.85 [0.16-21.69]
Frozen gait	8 / 24 (33)	12 / 23 (52)	3 / 25 (12)	9 / 20 (45)	0.096, 3.67 [0.84-16.03]	0.763, 1.33 [0.40-4.44]
Short steps gait	11 / 23 (48)	16 / 21 (76)	7 / 26 (27)	14 / 24 (58)	0.151, 2.49 [0.76-8.19]	0.342, 2.29 [0.63-8.31]
Postural instability or falls	20 / 31 (65)	27 / 30 (90)	12 / 26 (49)	24 / 27 (89)	0.190, 2.12 [0.73-6.16]	1.000, 1.13 [0.21-6.11]
Dystonia	9 / 30 (30)	11 / 26 (42)	11 / 28 (39)	15 / 26 (58)	0.582, 0.66 [0.22-1.97]	0.406, 0.54 [0.18-1.62]
with asymmetric presentation	*6 / 30 (20)*	*8 / 26 (31)*	*11 / 28 (39)*	*15 / 26 (58)*	*0.151, 0.39 [0.12-1.25]*	*0.093, 0.33 [0.10-1.02]*
Tremor	7 / 30 (23)	11 / 22 (37)	6 / 29 (21)	6 / 28 (21)	1.000, 1.17 [0.34-4.01]	**0.042*, 3.67 [1.07-12.55]**
Myoclonus	4 / 31 (13)	7 / 28 (25)	6 / 30 (20)	11 / 30 (37)	0.508, 0.59 [0.15-2.35]	0.402, 0.58 [0.19-1.79]
with asymmetric presentation	*3 / 31 (10)*	*4 / 28 (14)*	*4 / 30 (13)*	*7 / 30 (23)*	*0.707, 0.70 [0.14-3.41]*	*0.508, 0.55 [0.14-2.12]*
Cognitive impairment (general)	19 / 30 (63)	27 / 30 (90)	19 / 32 (59)	28 / 29 (97)	0.799, 1.18 [0.22-3.29]	0.612, 0.32 [0.03-3.28]
Executive dysfunction	17 / 27 (63)	21 / 25 (84)	17 / 22 (77)	21 / 21 (100)	0.358, 0.50 [0.14-1.77]	0.114, 0.11 [0.01-2.19]
Behavioral changes	9 / 28 (32)	14 / 25 (56)	8 / 27 (30)	11 / 26 (42)	1.000, 1.13 [0.36-3.54]	0.406, 1.74 [0.57-5.26]
Personality change	6 / 25 (24)	8 / 24 (33)	9 / 30 (30)	10 / 28 (36)	0.764, 0.74 [0.22-2.45]	1.000, 0.90 [0.29-2.84]
Lack of insight	8 / 26 (31)	11 / 25 (44)	4 / 22 (18)	7 / 22 (32)	0.505, 2.00 [0.51-7.84]	0.549, 1.68 [0.51-5.56]
Irritability	5 / 29 (17)	8 / 30 (27)	4 / 26 (15)	7 / 25 (28)	1.000, 1.15 [0.27-4.82]	1.000, 0.94 [0.28-3.07]
Hallucination	3 / 29 (10)	5 / 27 (19)	2 / 27 (7)	1 / 24 (4)	1.000, 1.44 [0.22-9.37]	0.195, 5.23 [0.56-48.38]
Limb apraxia	8 / 28 (29)	10 / 21 (48)	16 / 27 (59)	19 / 26 (73)	**0.031*, 3.64 [1.18-11.18]****	0.130, 0.33 [0.10-1.13]
Cortical sensory loss	3 / 20 (15)	4 / 19 (21)	6 / 19 (32)	7 / 18 (39)	0.273, 0.38 [0.08-1.82]	0.295, 0.42 [0.10-1.79]
with asymmetric presentation	*3 / 20 (15)*	*4 / 19 (21)*	*6 / 19 (32)*	*7 / 18 (39)*	*0.273, 0.38 [0.08-1.82]*	*0.295, 0.42 [0.10-1.79]*
Alien limb	0 / 29 (0)	2 / 27 (7)	2 / 22 (9)	4 / 21 (19)	0.181, 0.14 [0.01-3.05]	0.383, 0.34 [0.06-2.07]
with asymmetric presentation	0 / 29 (0)	0 / 27 (0)	0 / 22 (0)	0 / 21 (0)	*1.000, 0.76 [0.01-39.94]*	*1.000, 0.78 [0.01-41.03]*
Visuospatial deficits	2/ 19 (11)	2 / 15 (13)	5/ 19 (26)	7 / 16 (44)	0.405, 0.33 [0.06-1.96]	0.113, 0.20 [0.03-1.18]
Effortful, agrammatic speech	1 / 26 (4)	13/ 22 (14)	3 / 22 (14)	7/ 17 (41)	0.320, 0.25 [0.02-2.63]	0.341, 2.06 [0.57-7.47]
Impaired grammar/ sentence comprehension with relatively preserved wingle word comprehension	2 / 26 (8)	7 / 21 (33)	1 / 23 (4)	9/ 19 (47)	1.000, 1.83 [0.16-21.66]	0.520, 0.56 [0.15-1.99]
Groping, distorted speech production	4 / 27 (15)	8 / 21 (38)	5 / 23 (22)	8 / 20 (40)	0.715, 0.63 [0.15-2.12]	1.000, 0.92 [0.26-3.24]
Supranuclear vertical gaze palsy or decreased velocity of vertical saccades	13 / 26 (50)	15 / 24 (63)	10 / 30 (33)	22 / 29 (76)	0.278, 2.00 [0.68-5.89]	0.372, 0.53 [0.16-1.74]
Urinary incontinence	11 / 29 (38)	22 / 26 (85)	10 / 28 (36)	23 / 27 (85)	1.000, 1.10 [0.37-3.23]	1.000, 0.96 [0.21-4.30]
Speech and language impairment	12 / 28 (43)	21 / 28 (75)	18 / 28 (64)	24 / 30 (80)	0.180, 0.42 [0.14-1.22]	0.757, 0.75 [0.22-2.59]
Dysarthria	8 / 29 (28)	17 / 27 (63)	16 / 27 (59)	20 / 26 (77)	**0.030*, 3.82 [1.25-11.68]****	0.372, 0.51 [0.15-1.70]
Slurred speech	3 / 30 (10)	8 / 25 (32)	6 / 26 (23)	5 / 23 (22)	0.277, 0.37 [0.08-1.66]	0.523, 1.69 [0.46-6.21]
Pyramidal sign	7 / 30 (23)	11 / 27 (41)	16 / 31 (52)	18 / 30 (60)	**0.034*, 3.50 [1.17-10.54]****	0.189, 0.46 [0.16-1.32]

Data are presented as *n* (%), CBD: corticobasal degeneration, *P < 0.05 (Fisher's exact test); OR: odds ratio; 95% CI: 95% confidence intervals, OR [95%CI]**: OR [95%CI] (CBD mimics vs CBD); **Bold number indicates statistically significant.**

Supplementary table 5. Initial and final clinical diagnosis in patients with CBD

Diagnosis	CBD *n* = 32 Initial diagnosis	Final diagnosis
CBS/ CBD	7 (22)	16 (50)
PSP	6 (19)	8 (25)
Parkinson's disease	3 (9)	1 (3)
Dementia with Lewy bodies	1 (3)	1 (3)
Parkinsonian syndrome	1 (3)	
AD	4 (13)	3 (9)
FTD	2 (6)	3 (9)
Pick disease	1 (3)	
Dementia	2 (6)	
Myoclonus-dystonia syndrome	1 (3)	
Bipolar disorder	1 (3)	
Cerebral infarction	2 (6)	3 (9)
Idiopathic normal pressure hydrocephalus	1 (3)	3 (9)
Sequalae of subarachnoid hemorrhage		1 (3)

Data are presented as *n* (%), AD: Alzheimer disease; CBS/ CBD: corticobasal syndrome/ corticobasal degeneration; FTD: frontotemporal dementia; PSP: progressive supranuclear palsy

Supplementary Table 6. Pathological information in patients with CBD mimics

J-VAC ID number for CBD mimics	sex	Age at onset (y)	Age at death (y)	Disease duration (y)	Pathological diagnosis (Background pathology)
1	m	69	74	5	PSP
2[3,4]	m	60	69	9	AD
3[5]	m	60	72	12	Non-specific changes
4[6]	m	48	54	6	DLB
5	m	67	74	7	AD
6	m	71	77	6	FTLD-TDP
7	m	77	86	9	PSP
8	m	73	80	7	PSP
9	m	86	93	7	AD
10	f	79	90	11	AD
11	m	73	76	3	PSP
12[7]	m	54	63	9	FTLD-FUS (BIBD)
13	m	63	65	2	Prion disease
14	f	70	77	7	PSP
15	f	57	64	7	Glioblastoma
16	f	63	71	8	PSP
17	f	61	68	7	PSP
18	f	67	74	7	PSP, glioblastoma
19	f	74	82	8	GGT Type II
20	m	64	76	12	PSP
21	f	76	89	13	Pick disease
22	m	55	62	7	AD
23	f	64	71	7	PSP
24	m	59	68	9	PSP
25	m	70	79	9	AD
26	m	33	48	15	FTLD-TDP
27	m	60	72	12	FTLD-TDP
28	f	73	77	4	GGT Type II
29	m	79	88	9	PSP
30	m	60	78	18	PSP
31	m	76	85	9	DLB
32	m	70	76	6	PSP

AD: Alzheimer disease; DLB: diffuse Lewy body disease; f: female; FTLD-FUS: frontotemporal dementia-fused in sarcoma; FTLD-TDP: frontotemporal degeneration-TDP43 pathology; GGT: globular glial tauopathy; m: male; PSP: progressive supranuclear palsy; y: years or years old

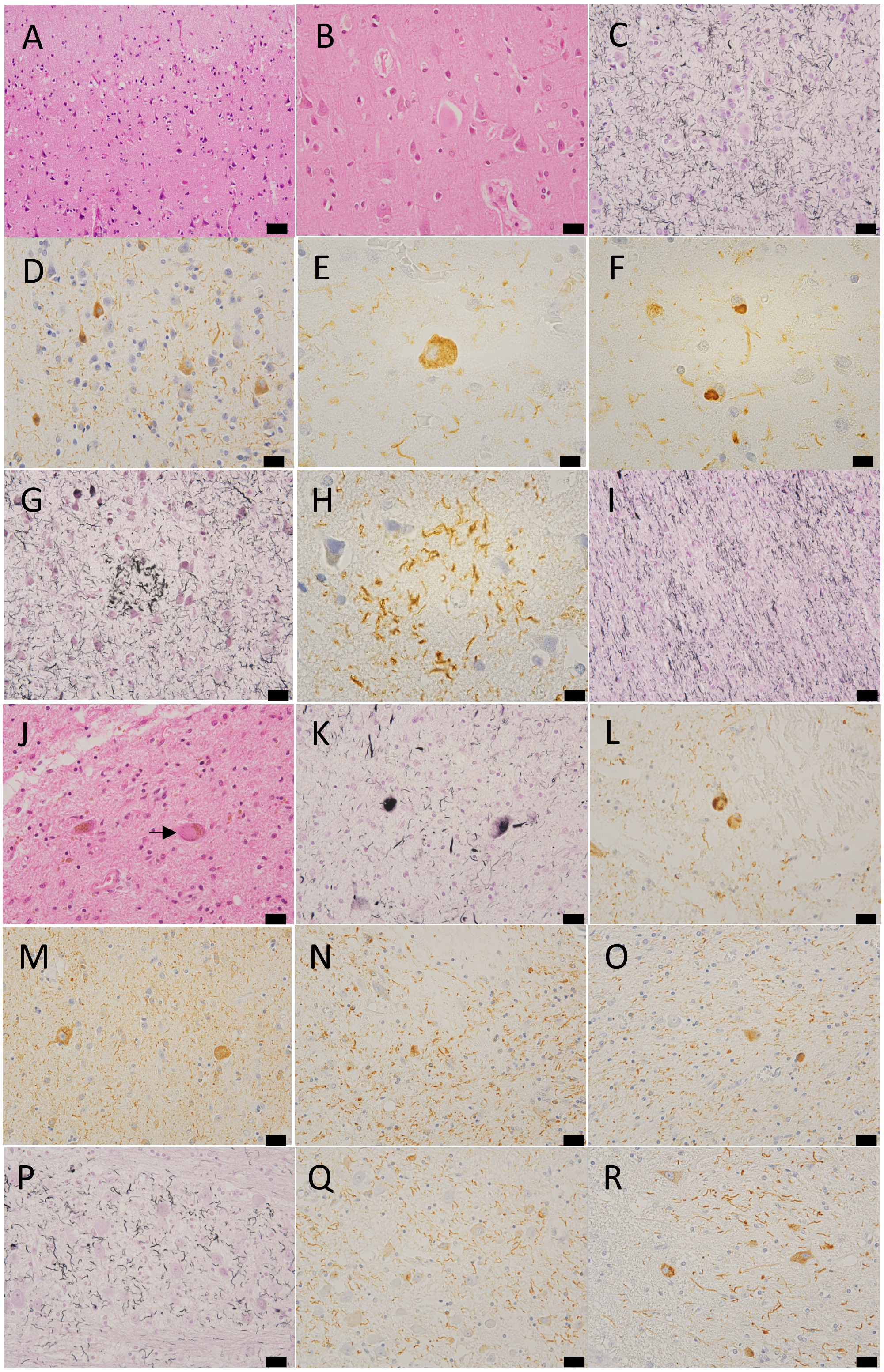

Supplementary Fig. 1 Neuropathological findings in patients with CBD.

Superficial spongiosis (**A**), ballooned neuron (**B**) and abundant threads and sparse neuronal inclusions (**C**) in the frontal cortex. (**D** and **E**) Pretangles and threads in the frontal cortex. (**F**) Small Pick body-like inclusions. (**G** and **H**) Astrocytic plaque and threads. (**I**) Numerous threads in the subcortical white matter. (**J**) Neuronal loss, gliosis, and corticobasal inclusion (arrow) in the substantia nigra. (**K** and **L**) Neuronal inclusions and threads in the substantia nigra. (**M**) Neuronal inclusions and threads in the putamen, pallidum interna (**N**) and subthalamic nucleus (**O**). (**P** and **Q**) Abundant threads and sparse neuronal inclusions in the pontine nucleus. (**R**) Pretangles and threads in the dentate nucleus. (**A, B** and **J**) hematoxylin and eosin stain, (**D-F, H, L-O, Q** and **R**) phosphorylated-tau immunohistochemistry and (**C, G, I, K** and **P**) Gallyas-Braak stain. (**A, H-O** and **R**) J-VAC ID number 20, (**B**) J-VAC ID number 13, (**C, D** and **G**) J-VAC ID number 18 and (**E, F, L, P, Q**) J-VAC ID number 19. Scale bars: (**A**) 50 µm, (**B-D, G, I-R**) 20 µm and (**E, F, H**) 10 µm. CBD: corticobasal degeneration; J-VAC: Japanese validation study of the consensus criteria for CBD diagnosis

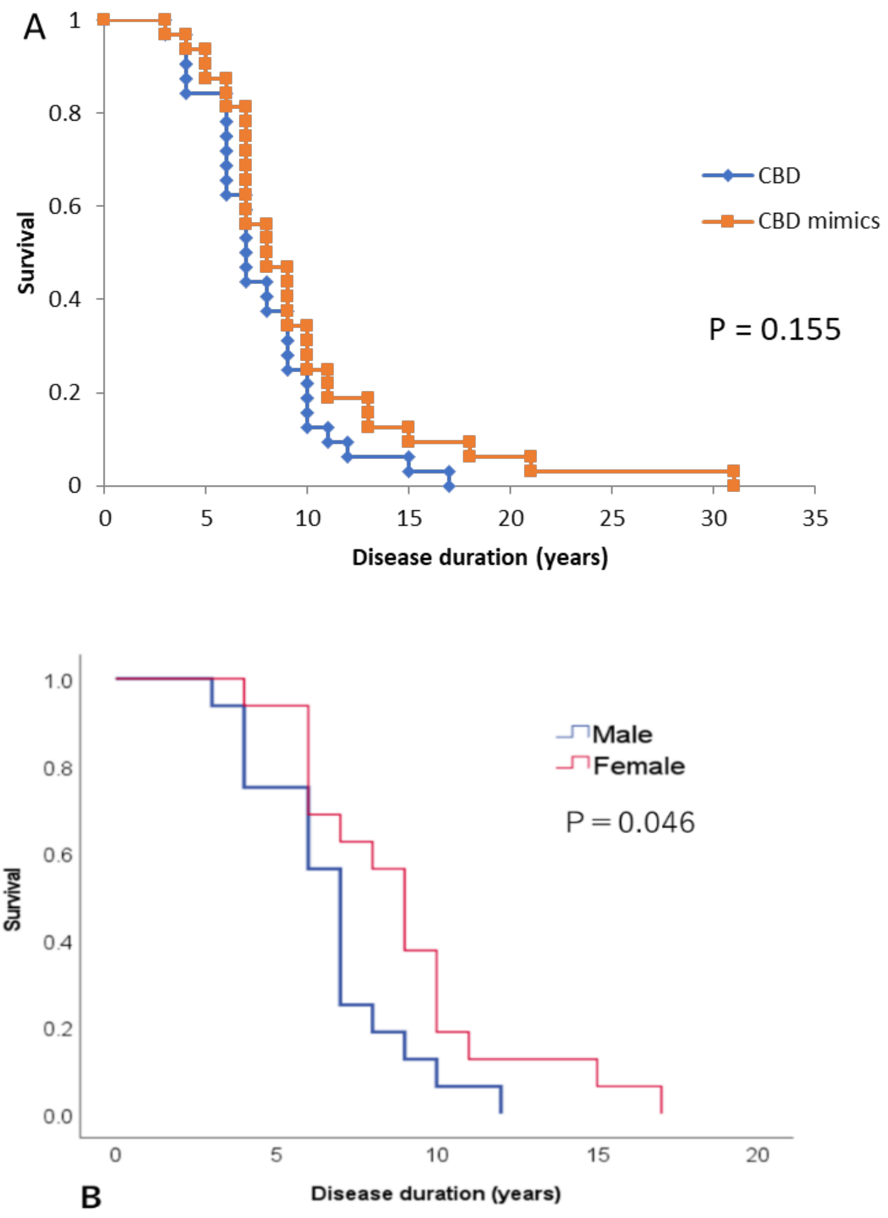

Supplementary Fig. 2 Kaplan–Meier survival curve in CBD and CBD mimics.

(A) The median survival time in patients with CBD (n =32) was 7.0 years, almost equal to that of CBD mimics (n = 32) (*P* = 0.155). **(B)** In patients with CBD, the survival time in women was significantly longer than those in men [7.0 years in men (n = 16), 9.0 years in women (n = 16), *P* = 0.046]. The log-rank test was used for comparisons. *P*-value < 0.05 were considered statistically significant. CBD: corticobasal degeneration

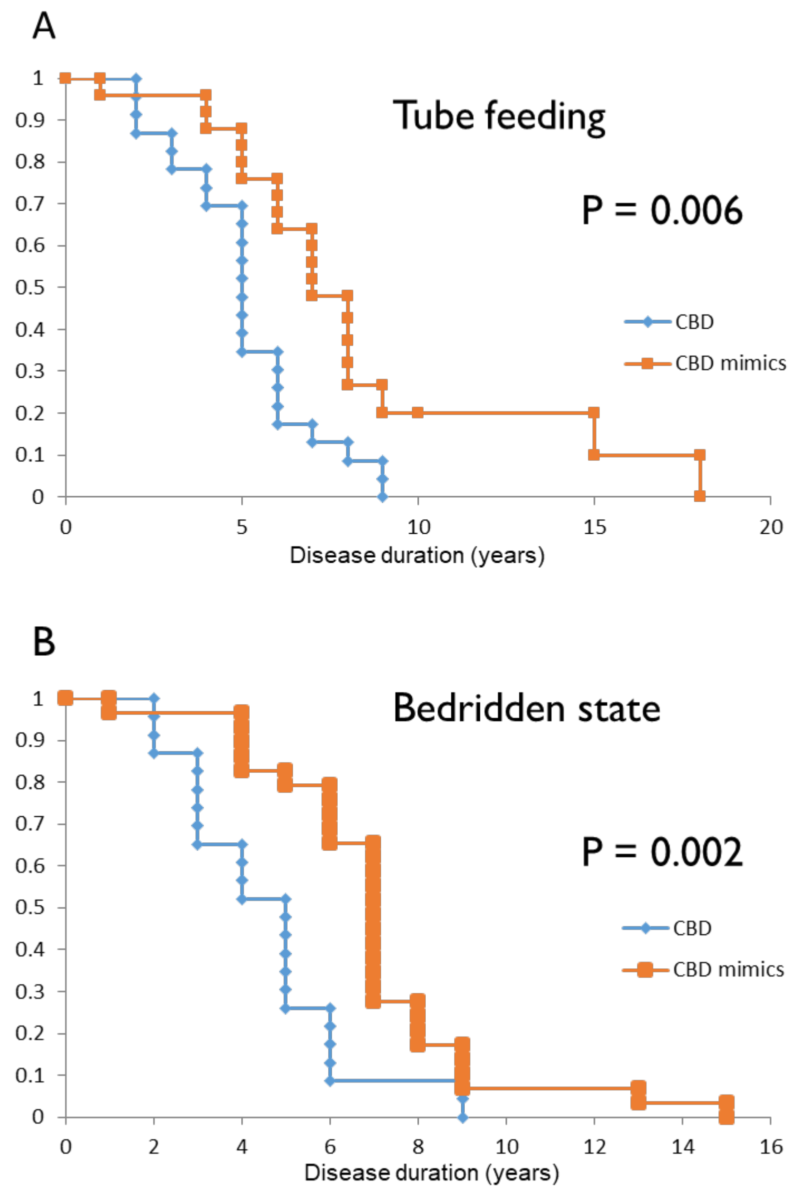

Supplementary Fig. 3 Interval to feeding or bedridden state in CBD compared with CBD mimics.

(A) Interval to tube feeding from the onset; **(B)** Interval to bedridden state from onset.

Both intervals to tube feeding and bedridden states from the onset of CBD (n = 32) were significantly earlier than those of CBD mimics (n = 32) (P = 0.006 and P = 0.002, respectively). The log-rank test was used for comparisons. P-value < 0.05 was considered statistically significant. CBD: corticobasal degeneration.

References

1) Mori K, Iwasaki Y, Ito M, Mimuro M, Yoshida M. [Decreased myocardial uptake of meta-iodobenzylguanidine in an autopsy-confirmed case of corticobasal degeneration with Lewy bodies restricted to the sympathetic ganglia]. *Rinsyo Shinkeigaku.* 2012;52:405-410 (Japanese)

2) Ishida C, Kato-Motozaki Y, Noto D, *et al.* An autopsy case of corticobasal degeneration with inferior olivary hypertrophy. *Neuropathology*. 2021;41:226-235.

3) Osako M, Mochizuki Y, Kugio Y, Mizutani T, Hayashi H. [Autopsy case of atypical type of Alzheimer's disease clinically diagnosed as corticobasal degeneration]. *Rinsyo Shinkeigaku*. 2007;47:581-584 (Japanese).

4) Homma T, Takubo H, Takahashi K, *et al.* Lateralized cortical involvement and contralateral parkinsonism without basal ganglia involvement in two autopsy cases of corticobasal syndrome-Alzheimer's disease. *J Alzhimers Dis*. 2014;40:51-55.

5) Yamaguchi S, Kojima H, Ohtake T, Oda M. Dementia of the frontal lobe type with clinicopathological features of corticobasal degeneration except for lack of glial cytoskeletal abnormalities. *Neuropathology*. 1999;19:196-202.

6) Ichinose K, Watanabe M, Mizutani S, Tanizawa T, Uchihara T, Fujigasaki H. An autopsy case of corticobasal syndrome with pure diffuse Lewy body disease. *Neurocase*. 2021;27:231-237.

7) Matsumoto A, Suzuki H, Fukatsu R, Shimizu H, Suzuki Y, Hisanaga K. An autopsy case of frontal lobar degeneration with the appearance of fused in sarcoma inclusions (basophilic inclusion body disease) clinical presenting corticobasal syndrome. *Neuropathology*. 2016;36:77-87.
