# Supplementary material for: Clinical course of pathologically confirmed corticobasal degeneration and corticobasal syndrome
Source: Brain Commun. 2023 Nov 3;5(6):fcad296. doi: 10.1093/braincomms/fcad296 (PMC10715783; doi:10.1093/braincomms/fcad296)
Supplement: fcad296_Supplementary_Data [file fcad296_supplementary_data.zip › Collaborative group of the J-VAC study@20230131.pdf]

Collaborative group of the J-VAC study

1	Michio Kobayashi	Department of Neurology, National Hospital Organization Akita National Hospital	84-40 Iwakiuchimichikawa-Idonosawa, Yurihonjo, Akita, 018-1393, Japan
2	Nobutaka Arai	Laboratory of Neuropathology, Tokyo Metropolitan Institute of Medical Science	Kamikitazawa 2-6-1, Setagaya-ku, Tokyo 156-8506、Japan
3	Koichi Wakabayashi	Department of Neuropathology, Hirosaki University Graduate School of Medicine	5,Zaifu-cho,Hirosaki-shi,Aomori, 036-8562,Japan
4	Ichiro Yabe	Department of Neurology, Faculty of Medicine and Graduate School of Medicine, Hokkaido University	Kita 15, Nishi 7, Kita-ku, Sapporo, Hokkaido, 060-8638 Japan
5	Satoshi Tanikawa	Institute for Chemical Reaction Design and Discovery (WPI-ICReDD), Hokkaido University	Kita 21, Nishi 10, Kita-ku, Sapporo, Hokkaido, 001-0021 Japan
6	Shinya Tanaka	Department of Cancer Pathology, Faculty of Medicine and Graduate School of Medicine, Hokkaido University & Institute for Chemical Reaction Design and Discovery (WPI-ICReDD), Hokkaido University	Kita 15, Nishi 7, Kita-ku, Sapporo, Hokkaido, 060-8638 Japan
7	Takanori Yokota	Department of Neurology and Neurological Sciences, Tokyo Medical and Dental University	1-5-45 Yushima, Bunkyo-ku, Tokyo 113-8519, Japan
8	Kinya Ishikawa	Department of Neurology and Neurological Sciences, Tokyo Medical and Dental University	1-5-45 Yushima, Bunkyo-ku, Tokyo 113-8519, Japan
9	Takuya Ohkubo	Department of Neurology and Neurological Sciences, Tokyo Medical and Dental University	1-5-45 Yushima, Bunkyo-ku, Tokyo 113-8519, Japan
10	Masato Hasegawa	Department of Brain & Neurosciences, Tokyo Metropolitan Institute of Medical Science	2-1-6 Kamikitazawa, Setagaya-ku, Tokyo, 156-8506, Japan
11	Yuko Saito	Department of Neuropathology (the Brain Bank for Aging Reseach),Tokyo Metropolitan Institute for Geriatrics and Gerontology	35-2 Sakae-cho, Itabashi-ku, Tokyo 173-0015, Japan
		Department of Pathology and Laboratory Medicine, National Center Hospital, National Center of Neurology and Psychiatry	4-1-1 Ogawa-Higashi, Kodaira,Tokyo 187-8551, Japan
12	Aya M Tokumaru	Department of Diagnostic Radiology, Tokyo Metropolitan Institute for Geriatrics and Gerontology	35-2 Sakae-cho, Itabashi-ku, Tokyo 173-0015, Japan
13	Renpei Sengoku	Department of Neurology and Neuropathology, Tokyo Metropolitan Institute for Geriatrics and Gerontology	35-2 Sakae-cho, Itabashi-ku, Tokyo 173-0015, Japan
14	Yasuhiro Sakashita	Department of Neurology and Neuropathology, Tokyo Metropolitan Institute for Geriatrics and Gerontology	35-2 Sakae-cho, Itabashi-ku, Tokyo 173-0015, Japan
15	Tomoyasu Matubara	Department of Neuropathology, Tokyo Metropolitan Institute for Geriatrics and Gerontology	35-2 Sakae-cho, Itabashi-ku, Tokyo 173-0015, Japan
16	Keita Sakurai	Department of Radiology, National Center for Geriatrics and Gerontology	7-430 Morioka-cho, Obu City, Aichi 474-8511, Japan
17	Takashi Komori	Department of Laboratory Medicine and Pathology (Neuropathology), Tokyo Metropolitan Neurological Hospital	2-6-1 Musashidai, Fuchu, Tokyo 183-0042,Japan
18	Keizo Sugaya	Department of Neurology, Tokyo Metropolitan Neurological Hospital	2-6-1 Musashidai, Fuchu, Tokyo 183-0042,Japan
19	Kentaro Hayashi	Department of Neurology, Tokyo Metropolitan Neurological Hospital	2-6-1 Musashidai, Fuchu, Tokyo 183-0042,Japan
20	Masaki Takao	Department of Laboratory Medicine, National Center Hospital, National Center of Neurology and Psychiatry	4-1-1 Ogawa-higashi-cho, Kodaira-shi, Tokyo 187-8551, Japan
21	Terunori Sano	Department of Laboratory Medicine, National Center Hospital, National Center of Neurology and Psychiatry	4-1-1 Ogawa-higashi-cho, Kodaira-shi, Tokyo 187-8551, Japan
22	Kazuko Hasegawa	Department of Neurology, NHO, Sagamihara National Hospital	18-1 Sakuradai Minami-ku Sagamihara City 252-0392, Japan
23	Yasushi Iwasaki	Department of Neuropathology, Institute for Medical Science of Aging, Aichi Medical University	1-1 Yazakokarimata, Nagakute, Aichi, 480-1195, Japan
24	Mari Yoshida	Department of Neuropathology, Institute for Medical Science of Aging, Aichi Medical University	1-1 Yazakokarimata, Nagakute, Aichi, 480-1195, Japan
25	Takayoshi Shimohata	Department of Neurology, Gifu University Graduate School of Medicine	1-1 Yanagido, Gifu City 501-1193, Japan
26	Yuichi Hayashi	Department of Neurology, Gifu University Graduate School of Medicine	1-1 Yanagido, Gifu City 501-1193, Japan
27	Keiko Mori	Department of Neurology, Oyamada Memorial Spa Hospital	5538-1 Yamadacho, Yokkaichi, Mie 512-1111, Japan
28	Masumi Ito	Department of Neurology, Oyamada Memorial Spa Hospital	5538-1 Yamadacho, Yokkaichi, Mie 512-1111, Japan
29	Motoko Sakai	Department of Neurology, NHO, Suzuka National Hospital	3-2-1 Kasado, Suzuka City, Mie Prefecture 513-8501, Japan
30	Harutoshi Fujimura	Department of Neurology, Osaka Toneyama Medical Center	5-1-1 Toneyama, Toyonaka, Osaka 560-8552, Japan
31	Kimiko Inoue	Department of Neurology, Osaka Toneyama Medical Center	5-1-1 Toneyama, Toyonaka, Osaka 560-8552, Japan
32	Chiaki Mori	Department of Neurology, Osaka Toneyama Medical Center	5-1-1 Toneyama, Toyonaka, Osaka 560-8552, Japan
33	Tomoko Saito	Department of Neurology, Osaka Toneyama Medical Center	5-1-1 Toneyama, Toyonaka, Osaka 560-8552, Japan
34	Takahiko Tokuda	Department Of Functional Brain Imaging , National Institutes for Quantum and Radiological Science and Technology (QST)	4-9-1 Anagawa, Inage-ku, Chiba-shi 263-8555, Japan
35	Ritsuko Hanajima	Division of Neurology, Department of Brain and Neurosciences, Faculty of Medicine, Tottori University	86 Nishi-cho,Yonago-shi,Tottori 683-8503, Japan
36	Hiroshi Takigawa	Division of Neurology, Department of Brain and Neurosciences, Faculty of Medicine, Tottori University	86 Nishi-cho,Yonago-shi,Tottori 683-8503, Japan
37	Tadashi Adachi	Division of Neuropathology, Department of Brain and Neurosciences, Faculty of Medicine, Tottori University	86 Nishi-cho,Yonago-shi,Tottori 683-8503, Japan
38	Kenji Nakashima	Department of Neurology, NHO, Matsue Medical Center	8-31 Agenogi, Matsue, Shimane 690-8556, Japan
39	Hisanori Kowa	Department of Neurology, NHO, Matsue Medical Center	8-31 Agenogi, Matsue, Shimane 690-8556, Japan
40	Osamu Yokota	Department of Psychiatry, Kinoko Espoir Hospital	2908 Higashi-ohto, Kasaoka 714-0071, Japan
		Department of Neuropsychiatry, Okayama University Graduate School of Medicine, Dentistry and Pharmaceutical Sciences	2-5-1 Shikata-cho, Okayama 700-8558, Japan
41	Seishi Terada	Department of Neuropsychiatry, Okayama University Graduate School of Medicine, Dentistry and Pharmaceutical Sciences	2-5-1 Shikata-cho, Okayama 700-8558, Japan
42	Hanae Nakashima-Yasuda	Department of Psychiatry, Zikei Hospital	100-2, Urayasu-honcho, Okayama, 702-8508, Japan
43	Tomoko Miki	Department of Psychiatry, Kinoko Espoir Hospital	2908 Higashi-ohto, Kasaoka 714-0071, Japan
		Department of Neuropsychiatry, Okayama University Graduate School of Medicine, Dentistry and Pharmaceutical Sciences	2-5-1 Shikata-cho, Okayama 700-8558, Japan
44	Chiho Ishida	Department of Neurology, NHO, Iou National Hospital	Ni 73-1 Iwademachi, Kanazawa, Ishikawa 920-0192 Japan
45	Yuko Kato-Motozaki	Department of Neurology, NHO, Iou National Hospital	Ni 73-1 Iwademachi, Kanazawa, Ishikawa 920-0192 Japan
46	Kiyonobu Komai	Department of Neurology, NHO, Iou National Hospital	Ni 73-1 Iwademachi, Kanazawa, Ishikawa 920-0192 Japan
47	Masaharu Tanaka	Department of Psychiatry, Mishima Hospital	1713-8 Fujikawa, Nagaoka, Niigata 940-2302, Japan
48	Jiro Idezuka	Department of Neurology, Ojiya Sakura Hospital	2732 Kowada, Ojiya, Niigata 947-0041, Japan
49	Osamu Onodera	Department of Neurology,Clinical Neuroscience Branch, Brain Research Institute, Niigata University	1-757 Asahimachidori, Chuo-ku Niigata 951-8585, Japan
50	Masato Kanazawa	Department of Neurology,Clinical Neuroscience Branch, Brain Research Institute, Niigata University	1-757 Asahimachidori, Chuo-ku Niigata 951-8585, Japan

51	Takeshi Ikeuchi	Department of Molecular Genetics, Brain Research Institute, Niigata University	1-757 Asahimachidori, Chuo-ku Niigata 951-8585, Japan
52	Akiyoshi Kakita	Department of Pathology, Brain Research Institute, Niigata University	1-757 Asahimachidori, Chuo-ku Niigata 951-8585, Japan
53	Hiroshi　Shimizu	Department of Pathology, Brain Research Institute, Niigata University	1-757 Asahimachidori, Chuo-ku Niigata 951-8585, Japan
54	Mari Tada	Department of Pathology, Brain Research Institute, Niigata University	1-757 Asahimachidori, Chuo-ku Niigata 951-8585, Japan
55	Yasuko Toyoshima	Department of Neurology, Brain Disease Center Agano Hospital	6317-15 Yasuda, Agano, Niigata 959-2221, Japan
		Department of Pathology, Brain Research Institute, Niigata University	1-757 Asahimachidori, Chuo-ku Niigata 951-8585, Japan
56	Kenju Aoki	Department of Neurology, Brain Disease Center Agano Hospital	6317-15 Yasuda, Agano, Niigata 959-2221, Japan
57	Masashi Aoki	Department of Neurology, Tohoku University Graduate School of Medicine	1-1, Seiryo-machi, Aoba-ku, Sendai, Miyagi, 980-8574, Japan
58	Takafumi Hasegawa	Department of Neurology, Tohoku University Graduate School of Medicine	1-1, Seiryo-machi, Aoba-ku, Sendai, Miyagi, 980-8574, Japan
59	Arifumi Matsumoto	Department of Neurology, National Hospital Organization Miyagi National Hospital	100 Kassenhara Aza, Takase Yamamotocho, Watarigun, Miyagi, 989-2202, Japan
60	Akio Kikuchi	Department of Occupational Therapy, Yamagata Prefectural University of Health Sciences	260 Kamiyanagi, Yamagata, Yamagata 990-2212, Japan
61	Toshiki Uchihara	Neurology Clinic With Neuromorphomics Laboratory, Nitobe-Memorial Nakano General Hospital	4-59-16 Chuo, Nakano-ku, Tokyo 164-8607, Japan
		Laboratory of Structural Neuropathology, Tokyo Metropolitan Institute of Medical Science	2-1-6 Kamikitazawa, Setagaya-ku, Tokyo 156-8506, Japan
62	Mutsufusa Watanabe	Department of Neurology, Tokyo Metropolitan Bokutoh Hospital	4-23-15 Koutoubashi, Sumida-ku, Tokyo, 130-8575,Japan
63	Masahisa Katsuno	Department of Neurology, Nagoya University Graduate School of Medicine	65 Tsurumai-cho, Showa-ku, Nagoya 466-8550, Japan
64	Hirohisa Watanabe	Department of Neurology, Fujita Health University School of Medicine	1-98 Dengakugakubo, Kutsukake-cho, Toyoake, Aichi 470-1192, Japan
65	Atsushi Hashizume	Department of Clinical Research Education, Nagoya University Graduate School of Medicine	65 Tsurumai-cho, Showa-ku, Nagoya 466-8550, Japan
66	Hisayoshi Niwa	Department of Neurology, Kariya Toyota General Hospital	5-15 Sumiyoshicho, Kariya, Aichi 448-8505, Japan
67	Keizo Yasui	Department of Neurology, Japanese Red Cross Aichi Medical Center Nagoya Daini Hospital	2-9 Myoken-cho, Showa-ku, Nagoya, Aichi 466-8650, Japan
68	Keita Ito	Department of Neurology, Hekinan Municipal Hospital	3-6 Heiwamachi, Hekinan, Aichi 447-8502, Japan
69	Tosiaki Ieda	Department of Neurology, Yokkaichi Municipal Hospital	2-2-37, Shibata, Yokkaichi-shi, Mie 510-8567, Japan
70	Yukihiko Washimi	Department of Geriatrics and Gerontology, National Center for Geriatrics and Gerontology	7-430 Morioka-cho, Obu City, Aichi 474-8511, Japan
71	Eiichiro Mukai	Department of Neurology, Aichi-pref Saiseikai Rehabilitation Hospital	1-1-18 Sako, Nishi-ku, Nagoya, Aichi 451-0052, Japan
72	Tatsushi Toda	Department of Neurology, Graduate School of Medicine, The University of Tokyo	7-3-1, Hongo, Bunkyo-ku, Tokyo 113-8655, Japan
73	Meiko Hashimoto Maeda	Department of Neurology, Graduate School of Medicine, The University of Tokyo	7-3-1, Hongo, Bunkyo-ku, Tokyo 113-8655, Japan
74	Akatsuki Kubota	Department of Neurology, Graduate School of Medicine, The University of Tokyo	7-3-1, Hongo, Bunkyo-ku, Tokyo 113-8655, Japan
75	Shigeo Murayama	Brain Bank for Neurodevelopmental, Neurological and Psychiatric Disorders, United Graduate School of Child Developmment ＆ Department of Neurology, Graduate School of Medicine, Osaka University	2-2 Yamadaoka Suita-shi, Osaka-fu, 565-0871, Japan
		Department of Neurology and Neuropathology, Tokyo Metropolitan Institute for Geriatrics and Gerontology	35-2 Sakae-cho,Itabashi-ku, Tokyo, 173-0015, Japan
		Department of Neurology, Tokyo Medical University Hospital	6-7-1 Nishishinjuku, Shinjuku-ku, Tokyo 160-0023, Japan
76	Yufuko Saito	Department of Neurology, National Hospital Organization Higashinagoya National Hospital	5-101 Umemorizaka, Meito-ku, Nagoya 465-8620, Japan
77	Ikuko Aiba	Department of Neurology, National Hospital Organization Higashinagoya National Hospital	5-101 Umemorizaka, Meito-ku, Nagoya 465-8620, Japan
